# WDR81 regulates adult hippocampal neurogenesis through endosomal SARA-TGFβ signaling

**DOI:** 10.1038/s41380-018-0307-y

**Published:** 2018-12-07

**Authors:** Min Wang, Changyong Tang, Ruxiao Xing, Xuezhao Liu, Xiu Han, Yinghao Liu, Lei Wang, Chonglin Yang, Weixiang Guo

**Affiliations:** 1 State Key Laboratory for Molecular and Developmental Biology, Institute of Genetics and Developmental Biology, Chinese Academy of Sciences, Beijing, 100101 China; 2 Graduate School, University of Chinese Academy of Sciences, Beijing, 100093 China; 3 State Key Laboratory for Conservation and Utilization of Bio-Resources in Yunnan, Center for Life Sciences, School of Life Sciences, Yunnan University, Kunming, 650091 China

**Keywords:** Neuroscience, Stem cells

## Abstract

Adult hippocampal neurogenesis, a process considered important for hippocampal function, is regulated at multiple molecular levels. Mutations in the gene encoding the WD40 repeat-containing protein WDR81 are associated with neurological disorders, including cerebellar ataxia, mental retardation, quadrupedal locomotion syndrome (CAMRQ2), and microcephaly. In this study, we show that ablation of WDR81 in adult neural progenitor cells (aNPCs) markedly reduced adult hippocampal neurogenesis and impaired hippocampus-dependent learning. WDR81 suppresses endosomal PtdIns3P synthesis, likely by inhibiting the assembly of the PI3K-III complex. In the absence of WDR81, endosomal PtdIns3P levels are greatly elevated, leading to endosomal persistence of the PtdIns3P-binding protein SARA and consequently hyperactivation of SARA-dependent TGFβ signaling. Inhibition of PI3K-III activity or suppression of SARA-dependent TGFβ signaling markedly ameliorated the defective adult neurogenesis in WDR81-deficient mice. Taken together, these findings not only uncover the requirement for the WDR81–SARA–TGFβ axis in adult hippocampal neurogenesis, but also suggest that defective adult hippocampal neurogenesis contributes to the etiology of WDR81-related neurological diseases.

## Introduction

Normal brain function requires the precise control of neurogenesis, which is tightly regulated by combinatorial functions of extrinsic signals and intrinsic programs. Pathological disturbance of this process contributes to the symptomatology of many neurological disorders. In the adult mammalian brain, newborn granular neurons are continuously integrated into hippocampal circuitry throughout life, and the fine-tuning of this process is important for hippocampal function [1, 2]. Therefore, understanding the molecular mechanisms that regulate adult neurogenesis will not only shed light on the processes that govern the functional integrity of the adult brain, but will also provide valuable insights into neurological disorders.

The precise regulation of adult neural stem cell proliferation and differentiation requires the coordinated action of a variety of signaling molecules (e.g., receptors and ligands). Through endosomal trafficking, such signaling molecules are distributed in a spatio-temporal manner, thereby determining cell fate, neuronal polarity, migration, and axonal outgrowth and guidance [3, 4]. Endosome-associated signaling receptors can either be delivered to lysosomes for degradation and signal termination, or recycled back to the plasma membrane in a tightly regulated way. These events are primarily controlled by endosomal Rab GTPases and phosphoinositides [5–7]. The early endosome-specific phosphatidylinositol 3-phosphate (PtdIns3P) plays important roles in the endosome–lysosome pathway [7, 8]. PtdIns3P is generated by the class III PI3K (PI3K-III) complex consisting of VPS34, VPS15,and Beclin1 [5, 9]. PtdIns3P can be converted to the late endosome-specific PtdIns(3,5)P_2_ by the FYVE finger-containing phosphoinositide kinase PIKfyve, dephosphorylated by myotubularin family phosphatases, or degraded in the endosomal lumen [5–7]. Appropriate levels of endosomal PtdIns3P are crucial for endosomal trafficking, disruption of which is implicated in several human neurological disorders [10, 11]. Nevertheless, it remains largely unknown how PtdIns3P-dependent endosomal trafficking plays a role in adult neurogenesis.

Recently, we and others revealed that WDR91 and WDR81, two WD40 repeat-containing proteins, act in complex with Beclin1, a subunit of the PI3K-III complex, to inhibit endosomal PtdIns3P synthesis, thereby facilitating early-to-late endosome conversion in the endosome–lysosome pathway [12, 13]. We further demonstrated that WDR91 is a Rab7 effector that is recruited to endosomes by GTP-bound Rab7, and its brain-specific depletion leads to defective dendritic arborization and reduced brain size in mice [14]. In addition, we found that brain-specific *Wdr81* knockout mice died quickly after birth, with accumulated p62 bodies in the cortical and striatal neurons [15]. Importantly, *WDR81* mutations were initially reported in a consanguineous family suffering from cerebellar ataxia, mental retardation, and quadrupedal locomotion syndrome (CAMRQ2) [16], and later in patients with microcephaly [17]. However, it is unclear whether and how WDR81 regulates hippocampal neurogenesis in the adult mammalian brain.

In the present study, we investigated the requirement for WDR81 in adult hippocampal neurogenesis. We found that specific depletion of WDR81 in adult neural progenitor cells (aNPCs) reduced adult hippocampal neurogenesis and impaired hippocampus-dependent learning. Mechanistically, we demonstrated that WDR81 inhibits the activity of PI3K-III, possibly by preventing the assembly of the PI3K-III complex, leading to PtdIns3P-dependent endosomal persistence of SARA, a PtdIns3P-binding protein in the TGFβ signaling pathway. Endosomal persistence of SARA led to hyperactivation of SARA-dependent TGFβ signaling, which in turn suppressed adult hippocampal neurogenesis. These findings unveil a regulatory role for WDR81 and endosomal PtdIns3P in adult hippocampal neurogenesis.

## Materials and methods

### Mice

Mice were housed in the animal facility at the Institute of Genetics and Developmental Biology (IGDB), Chinese Academy of Sciences, on a 12-h reverse light/dark cycle with lights on at 0800. All procedures and husbandry were performed according to protocols approved by the Institutional Animal Care and Use Committee at IGDB. *WDR81*^*f/f*^ mice were bred onto the C57BL/6 background as described previously [15]. The inducible conditional *Wdr81* knockout mice were generated by crossing Nestin-CreER^T2^ [18] driver with Rosa26-CAG-DTR-EGFP (BCG-T0-0001, BIOCYTOGEN). All mice in the study were backcrossed to the C57BL/6 background for at least six generations. To induce recombination, mice (8–10 weeks old) received Tamoxifen (Sigma-Aldrich, T5648) daily for 5 days (180 mg/kg intraperitoneal injection, 30 mg/ml in 10% EtOH/sunflower oil, Sigma-Aldrich) based on a published procedure [19].

### Cell lines and transfection

*Wdr81* WT and KO Hela cells were cultured as described previously [12]. For plasmid DNA transfections, 2 μg of plasmid DNA and/or 2 μg of shRNA were used and transfection was performed with Lipofectamine 2000 (Invitrogen, 11668019) according to the manufacturer’s instructions.

### Isolation, culture, and in vitro analyses of adult NPCs

Adult neural stem/progenitor cells (aNPCs) used in this study were isolated from the DG of 8–10-week-old male *Wdr81*^*f/f*^ mice based on published methods [20]. aNPCs were maintained in DMEM/F-12 medium containing 20 ng/ml basic fibroblast growth factor (FGF-2, PeproTech, #K1606), 20 ng/ml epidermal growth factor (EGF, PeproTech, #A2306), 1% N2 supplement (GIBCO, #17502-048), 1% antibiotic–antimycotic (GIBCO, #15240062), and 2mM L-glutamine (GIBCO, #25030081) in a 5% CO_2_ incubator at 37 °C. Half of the medium was replaced every two days. For TGFβ treatment, aNPCs were treated with TGFβ1 (Peprotech, 10021) at a dosage of 1 ng/ml.

For deletion of the *Wdr81* gene in aNPCs, lentiviruses expressing Cre-GFP were added twice (once per day) under proliferation conditions for 2 days before the initiation of proliferation or differentiation assays. Lentiviruses expressing dCre-GFP were used as control. Cre-induced recombination was confirmed by using both western blotting and immunocytochemistry.

Proliferation and differentiation analyses were performed as described previously [19–21]. To study cell proliferation, aNPCs were dissociated with trypsin and plated on poly-L-ornithine/laminin-coated slides (Nunc, #154526) at a density of 50,000 cells/well in proliferation medium. In total, 5 μM 5-bromo-2’-deoxyuridine (BrdU, Sigma-Aldrich, B5002) was added into the culture medium for 6–8 h. aNPCs were then washed with PBS and fixed with 4% paraformaldehyde for 30 min at room temperature, followed by immunohistochemical analysis. To detect BrdU incorporation, fixed cells were pretreated with 1 M HCl for 30 min at 37 °C, washed with borate buffer, pH 8.5, for 30 min, and then subjected to immunocytochemistry analyses. For the differentiation assay, aNPCs were cultured in differentiation medium, DMEM/F12 (1:1), containing 5 μM forskolin (Sigma-Aldrich, #F-6886) and 1 μM retinoic acid (Sigma-Aldrich, #R-2625) for 3 days, then fixed with 4% paraformaldehyde for 30 min, washed with PBS for 30 min, and subjected to immunocytochemistry analyses.

Immunocytochemistry staining was carried out as described [19–22]. Briefly, aNPCs were preblocked using PBS containing 5% normal goat serum (VECTOR, #S-1000) and 0.1% Triton X-100 for 30 min, followed by overnight incubation with primary antibodies: chicken anti-GFP (1:1000, Invitrogen, A10263), mouse anti-Tuj1 (1:1000, Promega, G7121), rabbit anti-GFAP (1:1000, DAKO, Z0334), rat anti-BrdU (1:3000, Abcam, ab-6326), mouse anti-Nestin (1:1000, BD Biosciences, 556309), rabbit anti-WDR81 (1:500, Proteintech, 24874-1-AP), guinea pig anti-WDR81 (1:1000; generated as described previously [12]), rabbit anti-SARA (1:500, Proteintech, 14821-1-AP), rabbit anti-EEA1(1: 500, Cell Signaling, 3288 S), and rabbit anti-cleaved caspase-3 (1:500, Cell Signaling, #9661). After washing with DPBS, cells were incubated with secondary antibodies that included goat anti-chicken-488, goat anti-mouse Alexa Fluor 568 (1:500, Invitrogen, #A11031), goat anti-rabbit Alexa Fluor 647 (1:500, Invitrogen, #A21245), or goat anti-rat Alexa Fluor 568 (1:500, Invitrogen, #A11077), followed by counterstaining with the fluorescent nuclear dye 4’,6-dimidino-2’-phenylindole dihydrochloride (DAPI, Sigma-Aldrich, #B2261). The coverslips were mounted with polyvinyl alcohol (PVA) mounting medium with DABCO (Sigma-Aldrich, 10981) and stored in the cold and dark before analysis.

The numbers of marker-positive cells (BrdU^+^, Tuj1^+^, or GFAP^+^, and cleaved caspase-3^+^), as well as total Cre-GFP-infected cells (GFP^+^) were quantified using a Nikon-ECLIPSE 80i microscope with NIS-Elements, BR. 3.00 software. The percentage of differentiated cells was calculated as the number of marker-positive cells divided by the total number of DAPI- or GFP-positive cells.

### Recombinant lentivirus production

Lentivirus production was performed as described previously [21]. Briefly, lentiviral transfer vector DNA and packaging plasmid DNA were co-transfected into 293 T cells. The medium was collected and pooled at 40, 64, and 88 h, and then filtered through a 0.2-μm filter. Viruses were concentrated by ultracentrifuge at 19,000 rpm for 2 h at 20 °C using a SW27 rotor (Beckman). The virus particles were washed once with phosphate-buffered saline (PBS) and then resuspended in 150 μl of PBS.

### Recombinant retrovirus production and in vivo grafting

Retrovirus production was performed as described previously [22]. Briefly, viral transfer vector DNA and packaging plasmid DNA were transfected into cultured 293T cells using calcium phosphate methods. The medium containing lentivirus was collected at 40, 64, and 88 h post transfection, pooled, filtered through a 0.2-µm filter, and concentrated using an ultracentrifuge at 19,000 rpm for 2 h at 20 °C using a SW27 rotor (Beckman). The virus particles were washed once and then resuspended in 50 µl of PBS. We routinely obtained 1 × 10^9^ infectious viral particles /ml.

In vivo retrovirus grating was performed as described [22]. Briefly, 7−8-week-old C57B/L6 male mice were anesthetized with isofluorane, and virus (1 μl with titer greater than 5 × 10^8^/ml) was injected stereotaxically into the dentate gyrus (DG) using the following coordinates relative to bregma, caudal: −2.0 mm; lateral: + /−1.7 mm; ventral: −1.9 mm.

### 3-MA and LY2109761 administration

For cell treatment, 3-MA (5 mmol/ml in PBS) was added to aNPCs, which were then subjected to proliferation and differentiation assay.

Systemic administration of 3-MA (15 mg/kg in PBS) or LY2109761 (20 mg/kg in PBS) was performed by intraperitoneal injection into adult mice every other day for 28 days. For cell proliferation assays, the mice also received one BrdU injection (200 mg/kg), and were killed at 2 h after BrdU injection. For cell differentiation assays, the mice also received BrdU injections (50 mg/kg), twice per day for two days, and were killed at 4 weeks after the final BrdU injection.

### RNA isolation and real-time PCR

RNA isolation used Trizol (Invitrogen, 15596018) based on the manufacturer’s protocol. The first-strand cDNA was generated by reverse transcription with oligo (dT) primer or random hexamers (Promega, A5001). Standard RT-PCR was performed using GoTaq DNA polymerase (Promega, M3001). To quantify the mRNA levels using real-time PCR, aliquots of first-strand cDNA were amplified with gene-specific primers and SYBR Green PCR Master Mix (CWBIOTECH, CW0682A) using a Bio-Rad Real-Time PCR System (CFX96). The PCR reactions contained 20–40 ng of cDNA, 1X Universal Master Mix, and 300 nM of forward and reverse primers in a final reaction volume of 20 μl. The ratio of different samples was calculated by the data analysis software built in with the Bio-Rad Real-Time PCR System.

The sequences of primers used for PCR reactions are as follows:

p15:

Sense: 5′-CTGGAGATTGACTGCGGGTT-3′

Anti-sense: 5′-TTGGTGATCCCCTTGGCTTC-3′

p27:

Sense: 5′-CCTCATCCCTTGTCCCGACT-3′

Anti-sense: 5′-GAAGTTCTGCGACTGCACAC-3′

p21:

Sense: 5′-CAGGCACCATGTCCAATCCT-3′

Anti-sense: 5′’-TTTCGGCCCTGAGATGTTCC-3′

CyclinD1:

Sense: 5′-CCCCTTGGGGACATGTTGTT-3′

Anti-sense: 5′-GCTCCCTACTCTCAGGGTGA-3′

CDK4:

Sense: 5′-TGGAAACTCTGAAGCCGACC-3′

Anti-sense: 5′-TTCTCACTCTGCGTCGCTTT-3′

GAPDH:

Sense: 5′-AATGGGAAGCTTGTCATCAACG-3′

Anti-sense: 5′-GAAGACACCAGTAGACTCCACGACATA-3′

### In vitro PI3K complex activity assay

The in vitro PI3K complex activity assay was performed as described previously [12]. aNPCs were harvested and lysed with lysis buffer (50 mM Tris-HCl, pH 7.4, 150 mM NaCl, 1% Triton X-100, and protease inhibitor cocktail). Endogenous Vps34 was immunoprecipitated by antibody against Beclin1 (MBL, PD017). Immunoprecipitated beads were extensively washed with lysis buffer and further washed twice with reaction buffer (40 mM Tris–HCl pH 7.5, 20 mM MgCl_2_, and 1 mg/ml BSA). The beads were then incubated with 10 μg of sonicated phosphatidylinositol (Sigma, 79403) and 1 μl of ATP (10 mM) in 30 μl of reaction buffer for 30 min at room temperature. Conversion of ATP to ADP was measured with an ADP-GloTM Kinase Assay Kit by following the instructions provided by the manufacturer (Promega, V6930).

### ELISA analysis of PtdIns3P

Cell lysates were collected, and the amount of PtdIns3P was determined using a PtdIns3P ELISA kit (Echelon, K-3300) according to the manufacturer’s instructions.

### Tissue preparation and immunohistochemistry

Immunohistochemistry was performed as described previously [19, 21]. At 1, 7, 14, 28, or 56 days after the last TAM injection, mice were euthanized by intraperitoneal injection of Avertin and then transcardially perfused with saline followed by 4% PFA. Brains were dissected out, post-fixed overnight in 4% PFA, and then equilibrated in 30% sucrose. Forty micrometer brain sections were generated using a sliding microtome and stored in a −20 ^o^C freezer as floating sections in 96-well plates filled with cryoprotectant solution (glycerol, ethylene glycol, and 0.1 M phosphate buffer, pH 7.4, 1:1:2 by volume).

The tissue sections were pre-blocked with TBS++ (TBS containing 3% goat or donkey serum and 0.3% Triton X-100) for 1 h at room temperature, followed by incubation with primary antibodies diluted in TBS++ overnight at 4 ^o^C. After washing three times, sections were incubated with secondary antibodies for 1 h at room temperature. All sections were counterstained with a nuclear counter stain, DAPI.

The primary antibodies used were: chicken anti-GFP (1:1000; Invitrogen, A10263), rabbit anti-Ki67 (1:500, Lab Vision/NeoMarkers, #NCL-Ki67p), rabbit anti-cleaved caspase-3 (1:500, Cell Signaling, #9661), mouse anti-NeuN (1:500, Millipore, MAB377), goat anti-DCX (1:100, Santa Cruz Biotechnology, SC-8066), rabbit anti-S100β (1:1000, Dako, Z0334), mouse anti-GFAP (1:1000, Millipore, MAB5804), guinea pig or rabbit anti-WDR81 (1:500, generated as described previously [12]). Fluorescent secondary antibodies were used as follows: goat anti-mouse 568 (1:500, A11004, Invitrogen), goat anti-rabbit 647 (1:500, A21245, Invitrogen), donkey anti-goat 568 (1:500, A11057, Invitrogen), donkey anti-rabbit 647 (1:500, A31573, Invitrogen), goat anti-mouse 647 (1:500, A21235, Invitrogen), goat anti-rabbit 568 (1:500, A11011, Invitrogen), donkey anti mouse 647 (1:500, A31571, Invitrogen). After staining, sections were mounted, coverslipped, and maintained at 4 ^o^C in the dark until analysis.

### Quantification and fate mapping of GFP^+^ cells in the DG

For quantification of GFP^+^ cells, 1 in 12 serial sections starting at the beginning of the hippocampus (relative to bregma, −1.5 mm) to the end of the hippocampus (relative to bregma, −3.5 mm) were used [19]. Quantification of GFP^+^ cells and phenotypic quantification of GFP^+^ cells in the granule layer were performed using a Nikon-ECLIPSE 80i microscope with NIS-Elements, BR. 3.00 software.

### Western blotting and immunoprecipitation

Cells were lysed in ice-cold Triton X-100 buffer (20 mM Tris–HCl, pH 7.5, 100 mM NaCl, 1% Triton X-100, 1 mM phenylmethanesulfonylfluoride, PMSF) or RIPA buffer (20 mM Tris–HCl pH 7.5, 100 mM NaCl, 0.1% SDS, 0.5% sodium deoxycholate, 1 mM PMSF) containing Complete Protease Inhibitor Cocktail (Roche, 11697498001). Cell lysates were spun down at 12,000 rpm for 10 min. In total, 50 μg of supernatants were resolved on sodium dodecyl sulfate polyacrylamide gels (SDS-PAGE) and blotted with the indicated antibodies. Primary antibodies used were: rabbit anti-WDR81 (1:500, Proteintech, 24874-1-AP), rabbit anti-p27 (1:100, Cell Signaling, 3688 S), rabbit anti-p57 (1:1000, Ruiying Biological, RLT3492), rabbit anti-p21 (1:1000, Santa Cruz, SC-397), rabbit anti-cyclinD1 (1:1000, Cell Signaling, 2922 S), rabbit anti-CDK4 (1:100, Santa Cruz, SC-260), rabbit anti-Smad2 (1:1000, Cell Signaling, 5339 S), rabbit anti-pSmad2 (1:1000, Cell Signaling, 3101 S), rabbit anti-pSmad1/5/9 (1:1000, Cell Signaling, 13820 S), rabbit anti-Smad1/5/9 (1:1000, Abcam, ab66737), rabbit anti-SARA (1:1000, Proteintech, 14821-1-AP), rabbit anti-TGFbetaRII (1:1000, Cell Signaling, 11888 S), rabbit anti-SARA (1:1000, Proteintech, 14821-1-AP), rabbit anti-VPS15 (1:1000, Abcam, ab128903), rabbit anti-VPS34 (1:1000, Cell Signaling, 3811 S), rabbit anti-Beclin1 (1:1000, MBL, PD017), rabbit anti-p-AKT (1:1000, Cell Signaling, 9018 S), rabbit anti-AKT (1:1000, Epitomics, 1085 S) and mouse anti-β-actin (1:1000, Sigma, A5441). The β-actin was used as the loading control.

To test the direct interaction of two proteins tagged with different epitopes, the corresponding expression vectors were co-transfected into HEK293 cells. Forty-eight hours later, cells were lysed in regular IP buffer (20 mM Tris-HCl, pH 7.5, 100 mM NaCl, 1% NP40, 1 mM PMSF, 1% glycerol) containing Complete Protease Inhibitor Cocktail. Cell lysates were spun at 12,000 rpm for 10 min at 4 °C, and the supernatants were incubated with FLAG antibody (M2)-conjugated or HA antibody-conjugated beads (~5μg antibody for each sample) overnight at 4 °C. The beads were centrifuged and washed three times with IP buffer. Precipitated proteins were resolved by SDS-PAGE, blotted, and probed with indicated antibodies.

### The fear conditioning test

The fear conditioning test was performed as described with modification [23]. Mice were placed into a shock chamber and allowed to explore for 2 min. Then, a white noise tone (87 dB) sounded for 30 s (conditional stimulus or “CS”). During the last 1.5 s of the tone, mice received a mild footshock (0.5 mA) (unconditioned stimulus or “US”). 2 min later, the same tone-footshock (CS-US) combination was delivered again. This cycle was presented a total of three times with a 60-s interval. The context test was performed 24 h after the training. During the test, mice were placed back into the same training chamber, and monitored by an overhead camera in the chamber for 5 min. No stimuli were applied. Two-hours after the context test, the cue test was performed, in which colored plexiglass inserts were placed into the training chamber to hide the shock grid and to change the “context” of the chamber. Mice were then placed in the chamber and monitored by the overhead camera for 6 min, during which two CS (spaced the same way as in the training session) were given. In the fear conditioning test, all events were programmed and all data were recorded through the Startle and Fear conditioning system (Panlab) and Packwin software (V2.0.05).

### Novel object location test

The object recognition test was performed as described with modification [24]. Mice were habituated to an empty white chamber by allowing them to freely explore for 15 min. After 24 h, each mouse was rehabituated to the empty chamber for 1 min and then placed in a holding cage, whereas two identical objects were placed in the corners of the arena 7 cm from the walls. Mice were returned to the chamber for training and allowed to freely explore until they accumulated a total of 30 s exploring the objects (exploration recorded when the front paws or nose contacted the object). Mice were then removed from the chamber, immediately infused, and returned to their home cage. After 24 h, object recognition was tested by moving one of the objects to a novel location counterbalanced across mice. The mice were reintroduced into the chamber and recorded for 10 min via digital video. The time spent exploring each object in the novel location tests was measured by watching the recorded behavior. Scoring was done by using two stopwatches and collecting the exploratory time for each object simultaneously. Novel location preference is expressed as the percentage of time exploring the novel location out of the cumulative time spent exploring both objects.

### Open-field assay

Mice were placed in an unfamiliar arena with clear side walls (10 × 10 × 16 inch; RWD life Science) and were allowed to freely explore the arena for 20 min. They were returned to their home cages after the test. Their locomotor activity was tracked by photo beams preinstalled in the arena and then analyzed by Panlab SMART 3.0 Software.

### Dark/light exploration assay

The test was conducted in a two-chamber shuttle box with an opaque divider in the middle (RWD Life Science). Each chamber measured 17 × 17 × 33 cm. An opening measuring 6 × 6 cm was located at the center bottom of the divider. The walls of one chamber were made of black plexiglass (dark chamber) while those of the other chamber were transparent (light chamber). The ceiling of the box was made of a sheet of aluminum with a round hole measuring 1 cm in diameter over the center of the light chamber. Mice were habituated in the room for 1 h before the test with the room lights off. During the test, the room was not illuminated but the light compartment was lit by a 60 W bulb closely placed over the hole in the ceiling. During the test, a mouse was first placed into the dark chamber and allowed to freely travel between the chambers for 5 min. The time spent in the light chamber was scored.

### Statistical analysis

All experiments were performed and analyzed by the same experimenter, blind to the animals’ genotype or group treatment under assessment. Animals were assigned to groups according to their genotypes, and no randomization was applied. Sample sizes were determined by power analyses based on a previously published study [25]. Variables followed a Gaussian distribution as revealed by the D’Agostino–Pearson normality test. All percentages were arcsine-transformed before statistical analysis. All data are shown as mean with standard error of mean (mean ± SEM). Statistical comparisons between the two groups were made using two-tailed Student’s *t* test. For the in vivo cell fate analysis of WT and cKO mice at different time point after TAM injection, two-way ANOVA were used. For the one-sample *t* test, the treatment group was first normalized by the control group, and then the one-sample *t* test against a mean of one was used on the normalized values. Probabilities of *P* < 0.05 were considered as significant. For the in vitro analysis, the number of independent experiments is specified in the legend of each figure.

## Results

### WDR81 ablation in aNPCs reduces adult hippocampal neurogenesis

To investigate the requirement for WDR81 in adult hippocampal neurogenesis, we first examined the expression pattern of WDR81 in the dentate gyrus (DG) of adult hippocampus using cell type-specific markers. WDR81 expression was detected in both Sox2^+^GFAP^+^ type 1 and Sox2^+^GFAP^−^ type 2 neural stem cells (NSCs), as well as Tbr2^+^ progenitor cells, DCX^+^ immature neurons and NeuN^+^ mature granule neurons in the adult DG of hippocampus (Fig. S1). Next, we isolated adult neural stem/progenitor cells (aNPCs) from *Wdr81*^*f/f*^ mice and infected them with lentivirus co-expressing green fluorescence protein (GFP) with Cre recombinase (lenti-CreGFP) or truncated Cre lacking recombinase activity (lenti-dCreGFP). Infection with lenti-CreGFP, but not lenti-dCreGFP, led to ablation of WDR81 expression in *Wdr81*^*f/f*^ aNPCs (Fig. S2a and b). *Wdr81*^*f/f*^ aNPCs infected with lenti-CreGFP (*Wdr81*^*-/-*^ aNPCs) had a significantly lower percentage of BrdU^+^ cells (Fig. S2c and d) and produced fewer and smaller neurospheres (Fig. S2e–g) than those infected with lenti-dCreGFP (*Wdr81*^*+/+*^ aNPCs). We then investigated the requirement for WDR81 in aNPC differentiation in vitro. While similar numbers of GFAP^+^ astrocytes differentiated from *Wdr81*^*+/+*^ and *Wdr81*^−*/*−^ aNPCs, Wdr81^−*/*−^ aNPCs produced far fewer Tuj1^+^ neurons than *Wdr81*^*+/+*^ aNPCs (Fig. S2h–k). These data suggest that WDR81 deficiency inhibits proliferation and neuronal differentiation of aNPCs.

**Fig. 1:**
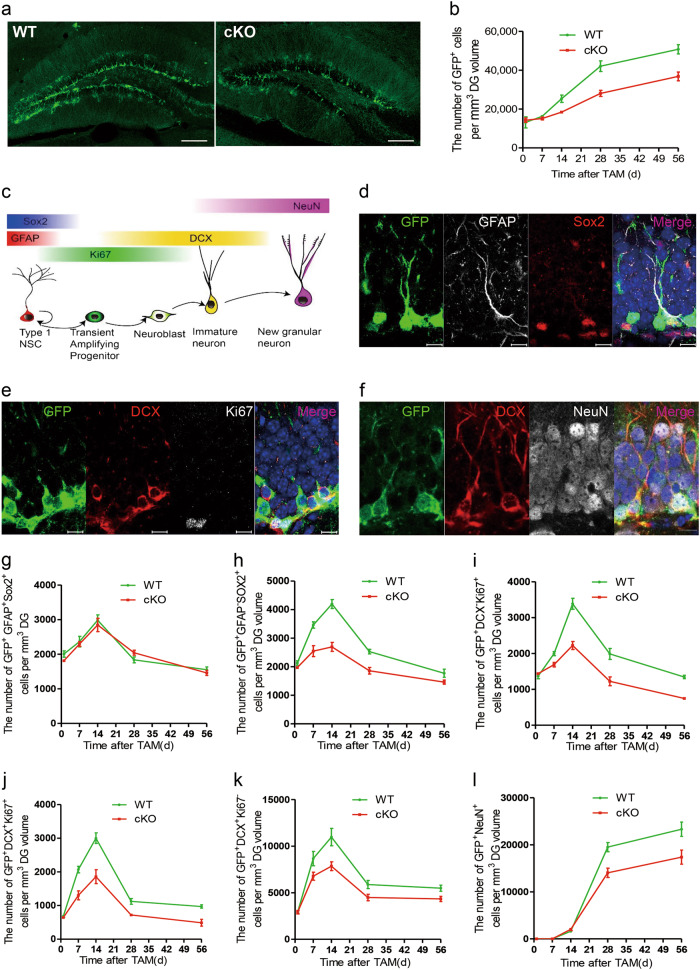
Deletion of *Wdr81* in Nestin-expressing cells inhibits neurogenesis in adult hippocampus. **a** Sample images of GFP^+^ cells in the DG of WT and *Wdr81 cKO* mice at 56 days after TAM injection. Scale bars, 200 µm. **b** Quantification of GFP^+^ cells in the DG of *Wdr81 cKO* mice and WT mice (WT vs. cKO, two-way ANOVA, F_1,20_ = 34.87, *p* < 0.0001). **c** Schematic diagram of cell lineage-specific markers during adult hippocampal neurogenesis. **d**–**f** Sample images used for fate mapping of GFP^+^ cells in the DG by co-staining of GFAP with Sox2, DCX with Ki67, or DCX with NeuN. Scale bars, 10 µm. **g**–**l** Quantification of GFP^+^GFAP^+^Sox2^+^ type 1 aNSCs (**g**, WT vs. cKO, 2-way ANOVA, F_1,20_ = 0.666, *p* = 0.424), GFP^+^GFAP^-^Sox2^+^ type 2 progenitor cells (h, WT vs. cKO, 2-way ANOVA, F_1,20_ = 83.94, *p* < 0.0001), GFP^+^DCX^−^Ki67^+^ transient amplifying progenitor cells (**i**, WT vs. cKO, 2-way ANOVA, F_1,20_ = 83.74, *p* < 0.0001), GFP^+^DCX^+^Ki67^+^ neuroblasts (**j**, WT vs. cKO, 2-way ANOVA, F_1,20_ = 67.21, *p* < 0.0001), GFP^+^DCX^+^Ki67^−^ immature neurons (**k**, WT vs. cKO, 2-way ANOVA, F_1,20_ = 23.61, *p* < 0.0001) and GFP^+^NeuN^+^ mature neurons (**l**, WT vs. cKO, 2-way ANOVA, F_1,20_ = 18.33, *p* = 0.0004) in the DG of *Wdr81 cKO* mice and WT mice. WT, *n* = 3 mice, cKO, *n* = 3 mice. Data are presented as mean ± SEM; *p < 0.05; **p < 0.01; ***p < 0.001

To further determine the role of WDR81 in neurogenesis in vivo, we generated inducible conditional *Wdr81* knockout mice (*Wdr81 cKO*) by crossing *Wdr81*^*f/f*^ mice with inducible Nestin-CreERT2 transgenic driver mice and Rosa26-stop-DTR-2A-EGFP reporter mice (Fig. S3a). Male littermates without the *Wdr81 flox* allele were used as wild-type controls (WT). After tamoxifen (TAM) injection, the expression of WDR81 was undetectable in GFP^+^ recombination cells in *Wdr81 cKO* mice (Fig. S3b). We then analyzed the number of GFP^+^ cells and their phenotypes in *Wdr81 cK*O and WT mice at 1, 14, 28, and 56 days after TAM injection to cover the critical development stages of adult hippocampal neurogenesis. In general, *Wdr81 cK*O mice generated significantly fewer GFP^+^ cells in the DG of adult hippocampus compared to WT mice (Fig. 1a, b). Next, we performed fate mapping of GFP^+^ cells using cell lineage markers to determine which cells were affected in *Wdr81 cKO* mice (Fig. 1c–f). At 1day after TAM injection, no difference was observed between WT and *Wdr81 cKO* mice in the number of GFP^+^ cells or their lineages (Fig. 1g–l). At all given time-points after TAM treatment, there was a similar number of GFP^+^GFAP^+^Sox2^+^ type 1 NSCs in WT and *Wdr81 cKO* mice (Fig. 1g). Starting from 7 days after TAM injection, however, *Wdr81 cKO* mice generated significantly fewer GFP^+^GFAP^−^Sox2^+^ type 2 NSCs than WT controls (Fig. 1h). Using the cell proliferation marker Ki67, we found that *Wdr81 cKO* mice had fewer proliferating GFP^+^DCX^−^Ki67^+^ progenitor cells than the WT controls from 7 days after TAM treatment (Fig. 1i). Subsequently, *Wdr81 cKO* mice produced fewer proliferating GFP^+^DCX^+^Ki67^+^ neuroblasts (Fig. 1j), GFP^+^DCX^+^Ki67^−^ post-mitotic DCX^+^ immature neurons (Fig. 1k), and GFP^+^NeuN^+^ mature neurons. (Fig. 1l). In contrast, the numbers of GFP^+^GFAP^+^S100β^+^ astrocytes were comparable in WT and *Wdr81 cKO* mice at all given time-points after TAM injection (Fig. S3c and d), which suggests that WDR81 is not required for astrocytic cell fate specification of aNPCs. Thus, WDR81 deficiency led to decreased proliferation of neural progenitor cells, and consequently to reduced neuronal differentiation in adult hippocampal neurogenesis. In addition, no elevation in caspase 3 activation was observed in *Wdr81*^*−/−*^ aNPCs, or their differentiated derivatives, or the GFP^+^ cells in *Wdr81 cKO* mice (Fig. S3e and f, Fig. S4). This excludes the involvement of apoptosis in this process.

### Deletion of WDR81 from aNPCs leads to impaired hippocampus-dependent learning

**Fig. 2:**
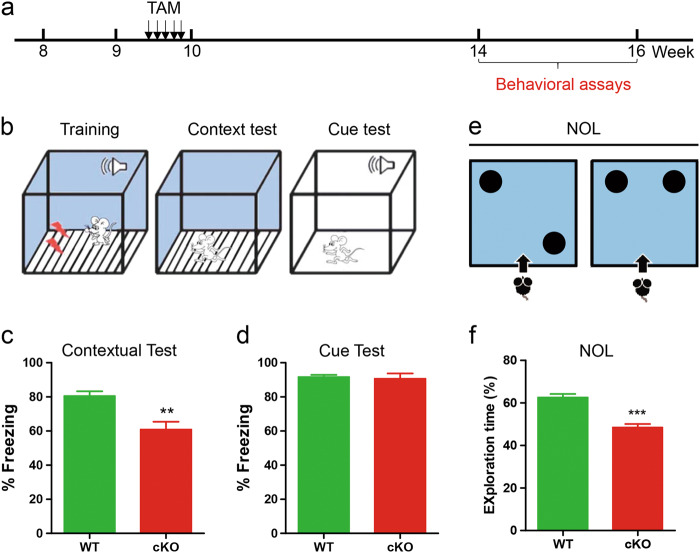
*Wdr81* deletion in Nestin-expressing cells impairs hippocampus-dependent learning. **a** Experimental scheme for hippocampus-dependent learning after TAM injection. **b**–**d** Analysis of the percentage of freezing behavior in WT and *Wdr81 cKO* mice during fear conditioning tests (**c**, the contextual test, WT vs. cKO, *t* test, *p* = 0.0037; **d**, the cue test, WT vs. cKO, *p* = 0.778. WT, *n* = 7 mice, cKO, *n* = 7 mice). **e** and **f** Analysis of the percentage of time spent exploring the newly-located object in WT and *Wdr81 cKO* mice (WT vs. cKO, *t* test, *p* < 0.001. WT, *n* = 10 mice, cKO, *n* = 13 mice). Data are presented as mean ± SEM; *p < 0.05; **p < 0.01; ***p < 0.001

Since hippocampal neurogenesis is involved in hippocampus-dependent learning, we generated another cohort of *Wdr81 cKO* mice by crossing *Wdr81*^*f/f*^ mice only with inducible nestin-CreERT2 transgenic driver mice (Fig. S5a) and assessed whether their learning ability is affected (Fig. 2a). Before performing the hippocampus-dependent learning tasks, we treated this cohort of mice with TAM for 5 days, and then labeled dividing cells with BrdU 1 week after TAM treatment to confirm that this cohort of *Wdr81 cKO* mice had impaired hippocampal neurogenesis (Fig. S5b). As expected, *Wdr81 cKO* mice displayed reduced numbers of BrdU^+^ cells (Fig. S5c and d), as well as BrdU^+^Sox2^+^GFAP^−^ type 2 NSCs (Fig. S5f and h), but not BrdU^+^Sox2^+^GFAP^+^ type 1 NSCs (Fig. S5e and g) at 2 h after BrdU injection. At 24 h after BrdU labeling, *Wdr81 cKO* mice displayed a significantly higher cell cycle exit index (Fig. S5i and j). Through in vitro cell cycle analysis, we found that *Wdr81*^*−/*−^ aNPCs indeed showed an increased number of cells in G1/G0 phase, but a decreased number in G2/M phase compared with *Wdr81*^*+/+*^ aNPCs (Fig. S6). At 4 weeks after BrdU exposure, *Wdr81 cKO* mice exhibited a decreased number of BrdU^+^NeuN^+^ neurons (Fig. S5k and l). These data were consistent with the in vivo cell lineage tracing. We therefore subjected this cohort of *Wdr81 cKO* and WT mice to a fear-conditioning test, in which performance in the context test is dependent on the function of both the hippocampus and the amygdala, while performance in the cue test is only dependent on the function of the amygdala [23]. *Wdr81 cKO* and WT mice had a similar level of freezing behavior in the cue test (Fig. 2b, d). However*, Wdr81 cKO* mice exhibited significantly less contextual learning as they showed a reduction of freezing behavior in the contextual test compared with WT mice (Fig. 2b, c). To further confirm that *Wdr81 cKO* mice have hippocampal learning deficits, we subjected the mice to the novel objective localization (NOL) test, which requires intact hippocampus function [24]. Compared with WT mice, *Wdr81 cKO* mice displayed no preference for the object in the novel location during NOL assessment (Fig. 2e, f). However, in comparison with control littermates, *Wdr81 cKO* mice did not change their overall level of ambulation level, quantified as the mean moving speed, moving distance, and moving time on the floor plane (Fig. S7a–c), nor did they show reduced exploration in the center of the arena, expressed as the number of entries into the center, and the time spent in the center (Fig. S7d–f). In addition, *Wdr81 cKO* mice did not show a decreased tendency to explore the light/dark compartments: the total number of entries and accumulated time spent exploring the light/dark compartments were not significantly different from their control littermates (Fig. S7g and h). Taken together, these behavioral assessments suggest that selective deletion of *Wdr81* in aNPCs leads to impaired hippocampus-dependent learning, but does not result in abnormal locomotor activity or overt anxiety-like behavior.

### WDR81 promotes neurogenesis by negatively regulating endosomal PtdIns3P levels in aNPCs

**Fig. 3:**
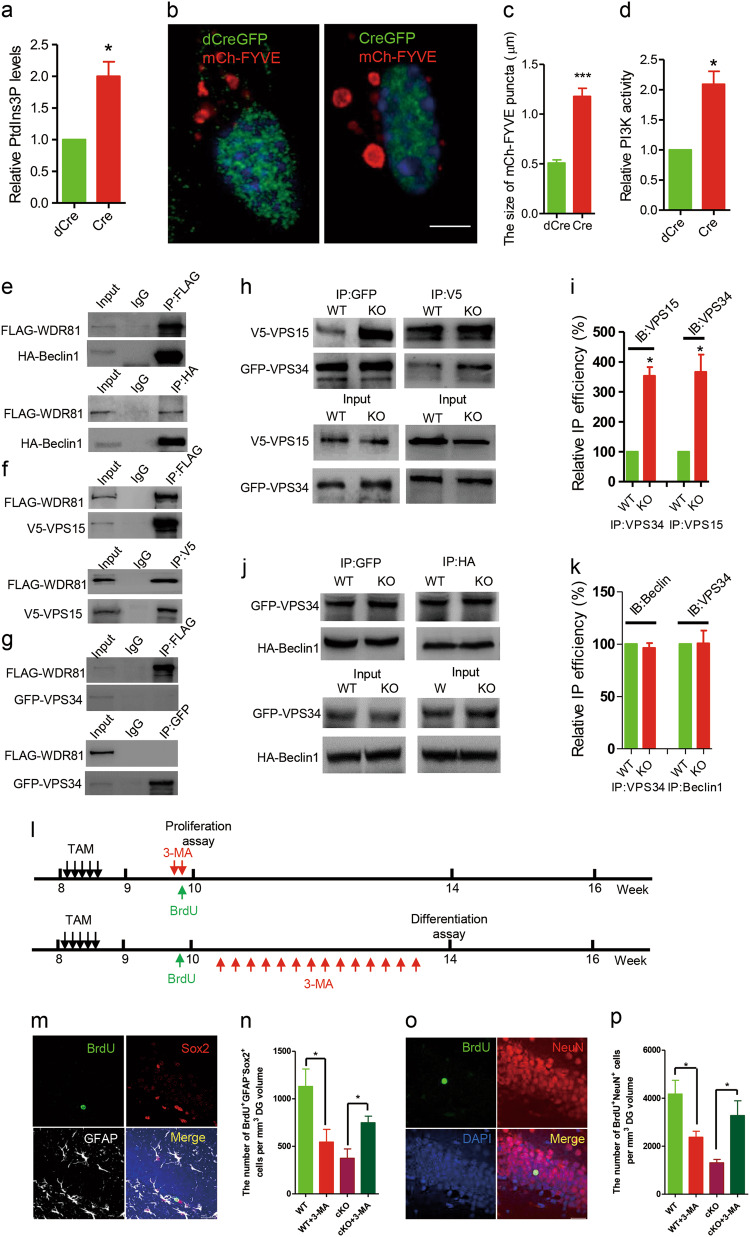
WDR81 suppresses PI3K-III activity in aNPCs. **a** Analysis of PI3K activity in *Wdr81*^*f/f*^ aNPCs infected with lenti-dCreGFP or lenti-CreGFP (dCre vs. Cre, *t* test, *p* = 0.049; dCre, *n* = 3 independent experiments, Cre, *n* = 3 independent experiments). **b** 2xFYVE-mCherry-positive endosomes in *Wdr81*^*f/f*^ aNPCs infected with lenti-CreGFP or lenti-dCreGFP. Scale bars, 5 µm. **c** Analysis of the size of 2xFYVE-mCherry endosomes in *Wdr81*^*f/f*^ aNPCs infected with lenti-dCreGFP or lenti-CreGFP (dCre vs. Cre, *t* test, *p* < 0.0001; *n* = 20 cells from three independent experiments). **d** PtdIns3P ELISA analysis of *Wdr81*^*f/f*^ aNPCs infected with lenti-dCreGFP or lenti-dCreGFP (dCre vs. Cre, *t* test, *p* = 0.036; dCre, *n* = 3 independent experiments, Cre, *n* = 3 independent experiments). **e**–**g** Co-IP of FLAG-WDR81 with HA-Beclin1, V5-VPS15, and GFP^−^VPS34. **h** and **i** Co-IP of VPS15 with VPS34 in WT and WDR81 KO cells (**h**) and quantification of the co-IP efficacy normalized to input (**i**, IP: VPS34, WT vs. KO, *t* test, *p* = 0.013; IP: VPS15, WT vs. KO, *t* test, *p* = 0.044; WT, *n* = 3 independent experiments, KO, *n* = 3 independent experiments). **j** and **k** Co-IP of Beclin1 with VPS34 in WT and WDR81 KO cells (**j**) and quantification of the co-IP efficacy normalized to input (**k**, IP: VPS34, WT vs. KO, *t* test, *p* = 0.514; IP: Beclin1, WT vs. KO, *t* test, *p* = 0.961, WT, *n* = 3 independent experiments, KO, *n* = 3 independent experiments). **l** Experimental scheme for assessing the proliferation and differentiation of aNPCs in the DG of adult hippocampus after TAM injection and 3-MA treatment. **m** Sample images of the DG stained with BrdU, Sox2, and GFAP at 2 h after BrdU injection. Scale bars, 20 µm. **n** Quantification of BrdU^+^GFAP^−^Sox2^+^ cells in the DG of *Wdr81 cKO* mice and WT mice treated with or without 3-MA at 2 h after BrdU injection (WT vs. WT + 3-MA, *t* test, *p* = 0.043; and cKO vs. cKO + 3-MA, *t* test, *p* = 0.014; WT, *n* = 5 mice, WT + 3-MA, *n* = 4 mice, cKO, *n* = 5 mice, cKO + 3-MA, *n* = 5 mice). **o** Sample images of the DG stained with BrdU and NeuN at 4 weeks after BrdU injection. Scale bars, 20 µm. **p** Quantification of BrdU^+^NeuN^+^ neurons in the DG of *Wdr81 cKO* mice and WT mice treated with 3-MA at 4 weeks after BrdU labeling (WT vs. WT + 3-MA, *t* test, *p* = 0.025; and cKO vs. cKO + 3-MA, *t* test, *p* = 0.012; WT, *n* = 5 mice, WT + 3-MA, *n* = 4 mice, cKO, *n* = 5 mice, cKO + 3-MA, *n* = 5 mice). Data are presented as mean ± SEM; *p < 0.05, ***p < 0.001

Because WDR81 is required to inhibit endosomal PtdIns3P [12], we next examined the endosomal PtdIns3P levels in *Wdr81*^*−/*−^ aNPCs. Using a Ptdlns3P ELISA assay, we found that *Wdr81*^−*/*−^ aNPCs had a significantly higher level of Ptdlns3P than *Wdr81*^*+/+*^ aNPCs (Fig. 3a). Moreover, endosomes labeled with mCherry-tagged 2xFYVE, which specifically binds to endosomal Ptdlns3P, were greatly enlarged in Wdr81^−*/*−^ aNPCs compared with *Wdr81*^*+/+*^ aNPCs (Fig. 3b, c). Consistent with this, PI3K-III activity was significantly higher in Wdr81^−*/*−^ aNPCs than in *Wdr81*^*+/+*^ aNPCs (Fig. 3d). By contrast, no obvious difference in the levels of p-AKT, an indicator for PI3K-I activity, was detected between *Wdr81*^*+/+*^ and *Wdr81*^*−/−*^ aNPCs (Fig. S9a and b). However, WDR81 was not co-localized with LC3-mCherry autophagosomes, and WDR81 deficiency did not affect general autophagy in Wdr81^−*/*−^ aNPCs (Fig. S8). Thus, WDR81 deficiency increased PI3K-III activity and hence endosomal PtdIns3P levels in aNPCs.

PI3K-III is a multimeric complex that consists of the VPS34 kinase, the scaffolding protein VPS15, and several accessory subunits, including Beclin1 [26]. The expression levels of VPS34, VPS15, and Beclin1 were comparable in *Wdr81*^*+/+*^ and *Wdr81*^*−/−*^ aNPCs (Fig. S9a and b). Therefore, we investigated whether WDR81 regulates PI3K-III activity by affecting the assembly of the PI3K-III complex. In co-immunoprecipitation (co-IP) assays, WDR81 co-precipitated with VPS15 and Beclin1, but not with VPS34 (Fig. 3e–g). In WDR81^−/−^ cells, a significant increase of the co-IP efficacy between VPS34 and VPS15 (Fig. 3h, i) was detected, while the interaction of VPS34 with Beclin1 was not changed (Fig. 3j, k). These results suggested that WDR81 deficiency enhances VPS15-VPS34 interaction, and subsequently increases the activity of VPS34.

To determine if the defective neurogenesis in *Wdr81*^*-/-*^ aNPCs results from the increase in endosomal PtdIns3P levels, we treated in vitro cultured aNPCs with 3-methyladenine (3-MA), a widely used PI3K-III inhibitor [27]. Compared with the vehicle (control) treatment, 3-MA caused a significant decrease of the percentage of BrdU^+^ cells and differentiated Tuj1^+^ cells (Fig. S9c–f). This is consistent with previous findings that PI3K-III activity is required for proliferation and differentiation of aNPCs [28]. Compared with the vehicle-treated *Wdr81*^*-/-*^ aNPCs, however, *Wdr81*^*-/-*^ aNPCs treated with 3-MA exhibited a significant increase in the population of both BrdU^+^ cells and Tuj1^+^ cells (Fig. S9c–f). In addition, 3-MA treatment rescued the cell cycle progression of WDR81-deficient aNPCs to the control levels (Fig. S10). These results indicated that reducing PI3K-III activity rescued the proliferation and differentiation defects of WDR81^-/-^ aNPCs in vitro. To prove this further, we assessed the effect of 3-MA treatment on aNPC proliferation in vivo by quantifying BrdU^+^ cells and BrdU^+^Sox2^+^GFAP^−^ type 2 NSCs in the DG of hippocampus 2 h after BrdU injection (Figure 3l). 3-MA-treated WT mice exhibited a significant decrease in the number of BrdU^+^ cells (Fig. S11a and b) and BrdU^+^Sox2^+^GFAP^−^ type 2 NSCs (Fig. 3m, n) compared with the vehicle-treated control. In contrast, the number of BrdU^+^ cells (Fig. S11a and b) and BrdU^+^Sox2^+^GFAP^−^ type 2 NSCs (Fig. 3m, n) was significantly increased in 3-MA-treated *Wdr81 cKO* mice. Similarly, 3-MA treatment caused a decrease in the number of BrdU^+^ cells (Fig. 3l and Fig. S11c and d) and BrdU^+^NeuN^+^ neurons (Fig. 3o, p) in WT mice, but significantly increased the number of BrdU^+^ cells (Fig. S11c and d) and BrdU^+^NeuN^+^ cells in *Wd81 cKO* mice (Fig. 3o, p) at 4 weeks after BrdU injection. Furthermore, 3-MA treatment significantly improved the hippocampus-dependent learning abilities of *Wdr81 cKO* mice in both the contextual fear conditioning test and the novel objective location test (Fig. S12). Altogether, these in vitro and in vivo data suggested that reducing PI3K-III activity has a beneficial effect on the abnormal hippocampal neurogenesis in *Wdr81 cKO* mice.

### WDR81 regulates endosomal SARA-TGFβ signaling

**Fig. 4:**
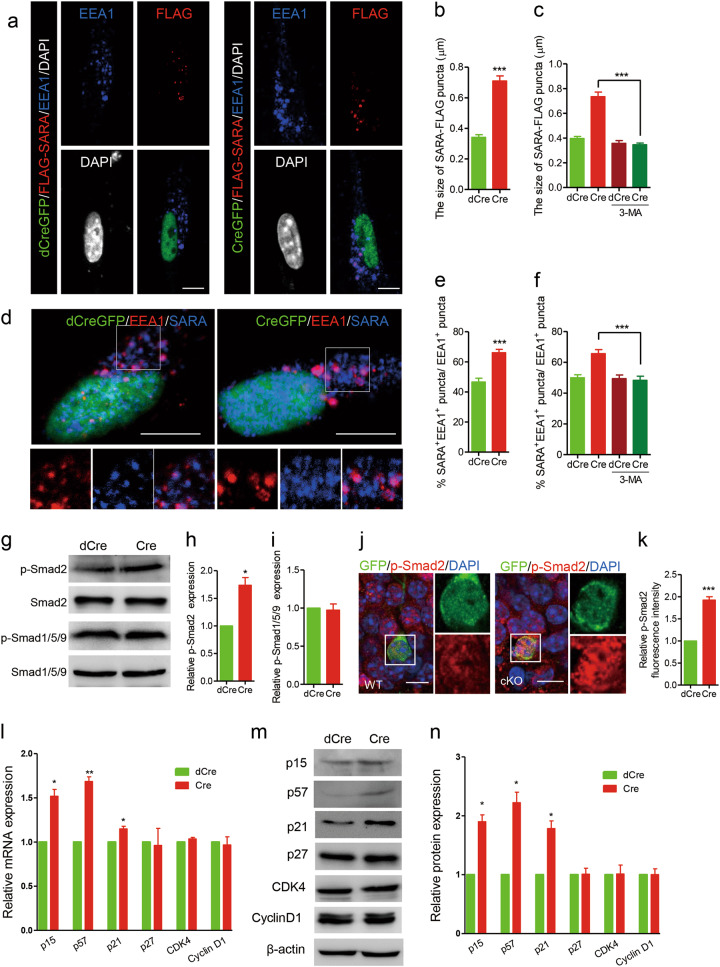
Elevated PI3K activity leads to endosomal persistence of SARA in WDR81-deficient aNPCs. **a** FLAG-SARA-positive puncta in *Wdr81*^*f/f*^ aNPCs infected with lenti-dCreGFP or lenti-CreGFP. Scale bars, 5 µm. **b** Analysis of the size of FLAG-SARA puncta in *Wdr81*^*f/f*^ aNPCs infected with lenti-dCreGFP or lenti-CreGFP (dCre vs. Cre, *t* test, *p* < 0.0001; *n* = 40 cells from three independent experiments). **c** Analysis of the size of FLAG-SARA-positive puncta in *Wdr81*^*f/f*^ aNPCs infected with lenti-dCreGFP or lenti-CreGFP and treated with 3-MA (Cre vs. Cre + 3-MA, *t* test, *p* < 0.0001; *n* = 40 cells from three independent experiments). **d** Co-staining of SARA with EEA1 in *Wdr81*^*f/f*^ aNPCs infected with either lenti-dCreGFP or lenti-CreGFP. Scale bars, 5 µm. **e** Analysis of the percentage of EEA1^+^ endosomes that are also positive for SARA (SARA^+^EEA1^+^) in *Wdr81*^*f/f*^ aNPCs infected with lenti-dCreGFP or lenti-CreGFP (dCre vs. Cre, t-test, *p* < 0.001, *n* = 15 cells from three independent experiments). **f** Analysis of the percentage of EEA1^+^ endosomes that are also positive for SARA (SARA^+^EEA1^+^) in *Wdr81*^*f/f*^ aNPCs infected with lenti-dCreGFP or lenti-CreGFP and treated with 3-MA (Cre vs. Cre + 3-MA, t-test, *p* < 0.001; *n* = 15 cells from three independent experiments). **g** Western blotting of p-Smad2, Smad2, p-Smad1/5/9, and Smad1/5/9 in *Wdr81*^*f/f*^ aNPCs infected with lenti-dCreGFP or lenti-CreGFP (dCre, *n* = 3, Cre, *n* = 3). **h** and **i** Quantification of p-Smad2 (dCre vs. Cre, *t* test, *p* = 0.0336) and p-Smad1/5/9 (dCre vs. Cre, *t* test, *p* = 0.7735) in *Wdr81*^*f/f*^ aNPCs infected with lenti-dCreGFP or lenti-CreGFP (dCre, *n* = 3 independent experiments, Cre, *n* = 3 independent experiments). **j** Immunohistological analysis of the expression of p-Smad2 in GFP^+^ cells in the DG of WT and *Wdr81 cKO* mice. Scale bars, 10 µm. **k** Quantification of the relative fluorescence intensity of p-Smad2 in GFP^+^ cells from WT and *Wdr81 cKO* mice (WT vs. cKO, *t* test, *p* < 0.0001; *n* = 20 cells from three mice). **l** Real-time PCR analyses of the expression of p-Smad2 target genes (p15, dCre vs. Cre, *p* = 0.021; p57, dCre vs. Cre, *p* = 0.005; and p21, dCre vs. Cre, *t* test, *p* = 0.034) and non-p-Smad2 target genes (p27, dCre vs. Cre, *t* test, *p* = 0.861; CDK4, dCre vs. Cre, *t* test, *p* = 0.167; and CyclinD1, dCre vs. Cre, *t* test, *p* = 0.757) in *Wdr81*^*f/f*^ aNPCs infected with either lenti-dCreGFP or lenti-CreGFP (dCre, *n* = 3 independent experiments, Cre, *n* = 3 independent experiments). **m**, **n** Western blot analyses (**m**) and quantification (**n**) of the expression of p-Smad2 target genes (p15, dCre vs. Cre, *t* test, *p* = 0.016; p57, dCre vs. Cre, *t* test, *p* = 0.02; and p21, dCre vs. Cre, *t* test, *p* = 0.026) and non-p-Smad2 target genes (p27, dCre vs. Cre, *t* test, *p* = 0.92; CDK4, dCre vs. Cre, *t* test, *p* = 0.93; and CyclinD1, dCre vs. Cre, *t* test, *p* = 0.97) in *Wdr81*^*f/f*^ aNPCs infected with either lenti-dCreGFP or lenti-CreGFP (dCre, *n* = 3 independent experiments, Cre, *n* = 3 independent experiments). Data are presented as mean ± SEM; *p < 0.05; **p < 0.01; ***p < 0.001

Given that WDR81 deficiency led to a significant increase in endosomal Ptdlns3P and consequently caused defective neurogenesis, we sought to determine the signaling pathway that is misregulated by the elevated PtdIns3P in *Wdr81*^*-/-*^ aNPCs. We firstly examined the gene expression levels of several FYVE-domain-containing proteins that are able to bind PtdIns3P and are implicated in signal transduction [29], including SARA (SMAD anchor for receptor activation), FGD1 (FYVE, RhoGEF, and PH domain containing 1) and Mtmr3 (myotubularin-related protein 3). Among them, SARA has the highest expression levels in aNPCs (Fig. S13a). In addition, SARA is located on endosomes (which are named SARA endosomes) and regulates cell division of NPCs in zebrafish spinal cord [30]. In *Wdr81*^*-/-*^ aNPCs, FLAG-tagged SARA was enriched on the abnormally enlarged endosomes (Fig. 4a, b), though the total protein levels of endogenous SARA were not changed (Fig. S13b and c). However, SARA did not co-precipitate with WDR81 (Fig. S13d). Importantly, 3-MA treatment fully rescued the enlargement of FLAG-SARA-positive endosomes in *Wdr81*^*-/-*^ aNPCs to the WT level (Fig. 4c, Fig. S14). This suggested that SARA probably persisted on the enlarged endosomes in *Wdr81*^*-/-*^ aNPCs. The percentage of EEA1^+^ endosomes that were also positive for SARA was indeed significantly higher in *Wdr81*^*-/-*^ aNPCs than in *Wdr81*^*+/+*^ aNPCs (Fig. 4d, e). This increase was rescued by 3-MA treatment (Fig. 4f).

SARA is believed to be required for the recruitment of Smad2 to the ligand-bound transforming growth factor-β receptor (TGFβR), an event that mediates downstream signal transduction [31, 32]. We therefore investigated whether TGFβ signaling is changed in *Wdr81*^*-/-*^ aNPCs. WDR81 deficiency led to attenuated degradation of TGFβR induced by TGFβ1 treatment in aNPCs (Fig. S13e and f). Consistent with this, the expression level of p-Smad2 (an indicator for activation of TGFβ signaling) was increased in *Wdr81*^*-/-*^ aNPCs, while the levels of p-Smad1/5/9, which indicate the activation of BMP signaling, remained similar to those in *Wdr81*^*+/+*^ aNPCs (Fig. 4g-i). In addition, immunofluorescence analysis of *Wdr81 cKO* mice also revealed an increase in p-Smad2 compared with WT mice (Fig. 4j and k). Accordingly, *Wdr81*^*-/-*^ aNPCs exhibited increased mRNA expression of Smad2 downstream target genes that trigger cell cycle exit of aNPCs [33, 34], such as p15, p57, and p21; the protein levels were also elevated (Fig. 4l–n). In contrast, the expression of non-Smad2 downstream target genes, such as p27, CDK4, and cyclin D1, was comparable in *Wdr81*^*+/+*^ and *Wdr81*^*-/-*^ aNPCs (Fig. 4l–n). Taken together, these data suggested that WDR81 deficiency impaired SARA-endosome trafficking, leading to prolonged SARA-mediated TGFβ signaling in aNPCs.

### Inhibition of SARA-mediated TGFβ signaling rescues the adult hippocampal neurogenesis defects in *Wdr81 cKO* mice

**Fig. 5:**
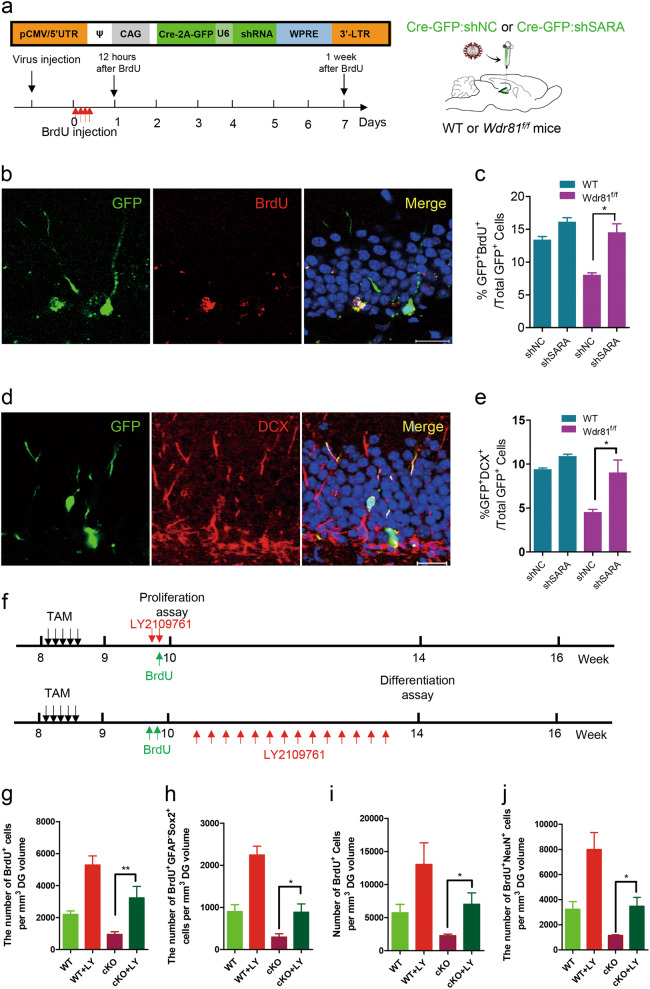
Inhibition of endosomal SARA-TGFβ signaling rescues the adult hippocampal neurogenesis defects in *Wdr81* cKO mice. **a** Schematic illustration of the retroviral vectors and the experimental timeline of in vivo retrovirus labeling for analyzing aNPC proliferation and differentiation in the adult DG. **b** Representative images of retrovirus-labeled GFP^+^ cells co-stained with BrdU in the adult DG. Scale bar = 50 μm. **c** Analysis of the number of BrdU^+^GFP^+^ cells in the DG of *Wdr81* cKO mice treated with retrovirus-mediated SARA shRNA (cKO + shNC vs. cKO + shSARA, *t* test, *p* = 0.043; shNC, *n* = 3 mice, shSARA, *n* = 3 mice). **d** Representative images of retrovirus-labeled GFP^+^ cells co-stained with DCX. Scale bar = 50 μm. **e** Quantification of DCX^+^GFP^+^ cells in the DG of *Wdr81* cKO mice treated with retrovirus-mediated SARA shRNA (cKO + shNC vs. cKO + shSARA, *t* test, *p* = 0.042; shNC, *n* = 3 mice, shSARA, *n* = 3 mice). **f** Experimental scheme for assessing the proliferation and differentiation of aNSCs in the DG of adult hippocampus after treatment with the TGFβ inhibitor LY2109761 (LY). **g**, **h** Quantification of BrdU^+^ cells (**g**, cKO vs. cKO + LY, *t* test, *p* = 0.008) and BrdU^+^GFAP^-^Sox2^+^ cells (h, cKO vs. cKO + LY, *t* test, *p* = 0.016) in the DG of *Wdr81 cKO* mice and WT mice treated with LY at 2 h after BrdU injection (WT, *n* = 5 mice, WT + LY, *n* = 6 mice, cKO, *n* = 6 mice, cKO + LY, *n* = 5 mice). **I,** **j** Quantification of BrdU^+^ cells (**i**, cKO vs. cKO + LY, *t* test, *p* = 0.033) and BrdU^+^NeuN^+^ cells (**j**, cKO vs. cKO + LY, *t* test, *p* = 0.014) in the DG of *Wdr81 cKO* mice and WT mice treated with LY at 4 weeks after BrdU injection (WT, *n* = 7 mice, WT + LY, *n* = 6 mice, cKO, *n* = 5 mice, cKO + LY, *n* = 6 mice). Data are presented as mean ± SEM; *p < 0.05; **p < 0.01

Since excessive activation of PI3K-III in *Wdr81*^*-/-*^ aNPCs led to persistent recruitment of SARA onto endosomes, and subsequent hyper-activation of SARA-mediated TGFβ signaling, we hypothesized that inhibition of SARA-mediated TGFβ signaling may rescue the adult neurogenesis deficits in *Wdr81 cKO* mice. Compared with the control shRNA treatment, shRNA knockdown of SARA in *Wdr81*^*-/-*^ aNPCs in vitro led to a significant increase in the percentage of BrdU^+^ cells and differentiated Tuj1^+^ cells (Fig. S15a–c). To confirm the specificity of shRNA manipulations, we generated lentiviruses expressing a fusion protein of FLAG and a shRNA-resistant form of SARA (SARA^R^) that harbors two silent mutations within the sequence targeted by shSARA (Fig. S15a). Western blot analysis confirmed the resistance of SARA^R^ to shSARA in vitro (Fig. S15a). In addition, re-expression of SARA^R^ eliminated the rescue effect of shSARA on aNPC proliferation and neuronal differentiation caused by WDR81 deficiency (Fig. S15b and c). SARA shRNA also downregulated the expression level of p-Smad2 in *Wdr81*^*-/-*^ aNPCs (Fig. S15d and e). Knockdown of SARA also rescued the cell cycle progression of WDR81-deficient aNPCs to the control levels (Fig. S16). We then stereotaxically injected retroviruses co-expressing Cre-2A-GFP and SARA shRNA (Fig. 5a) into the DG of adult hippocampus to inactivate WDR81 and SARA expression in aNPCs. Compared with the control shRNA injection, retrovirus-mediated SARA shRNA led to a significant increase in GFP^+^BrdU^+^ progenitor cells and GFP^+^DCX^+^ differentiated neurons in *Wdr81 cKO* mice (Fig. 5b–e). Thus, downregulation of SARA rescued the proliferation and differentiation defects of aNPCs caused by loss of WDR81.

Finally, we evaluated the rescuing effect of LY2109761 (LY), a small molecule inhibitor of the TGF-β receptor type 1/type II kinases, on hippocampal neurogenesis in *Wdr81* cKO mice (Fig. 5f). LY-treated WT mice exhibited an increased number of BrdU^+^ cells, BrdU^+^Sox2^+^GFAP^−^ type 2 NSCs, and BrdU^+^NeurN^+^ neurons compared with the vehicle treatment (Fig. 5g–j). This is consistent with the previous study showing that inhibition of TGFβ signaling promoted proliferation and neuronal differentiation of aNPCs [35]. Interestingly, LY treatment induced a significant increase in BrdU^+^ cells, BrdU^+^Sox2^+^GFAP^−^ type 2 NSCs and BrdU^+^NeurN^+^ neurons in *Wdr81 cKO* mice (Fig. 5g–j). Taken together, these data suggested that inhibition of SARA-mediated TGFβ signaling rescued the adult hippocampal neurogenesis defects caused by WDR81 deficiency.

## Discussion

Adult neurogenesis recapitulates the entire process of embryonic neural development and has been implicated in learning/memory and affective behaviors [1, 2, 36]. Using adult mouse hippocampal neurogenesis as a model, we showed that selective deletion of *Wdr81* in aNPCs markedly reduced hippocampal neurogenesis and impaired hippocampus-dependent learning. The interaction of WDR81 with VPS15 and Beclin1 likely inhibits the assembly of PI3K-III complex, thereby preventing endosomal PtdIns3P synthesis. As a result, WDR81 depletion in aNPCs led to elevated levels of endosomal PtdIns3P, which in turn led to abnormal neurogenesis in adult hippocampus. Supporting this conclusion, chemical inhibition, or shRNA inactivation of PI3K-III markedly ameliorated the defective neurogenesis in *Wdr81 cKO* mice. Together these results suggest that WDR81 promotes adult neurogenesis by controlling endosomal PtdIns3P levels in aNPCs (Fig. S17).

Although exhaustive efforts have been devoted to understanding the underlying mechanisms that regulate neurogenesis, significant questions still remain to be addressed regarding the molecular mechanisms regulating different aspects of neurogenesis under physiological and pathological conditions. PI3K signaling is a vital pathway controlling cell survival, proliferation, and apoptosis [37], and is involved in a number of neurological disorders, such as autism and schizophrenia [38]. Based on primary structure, regulation, and lipid substrate specification, the PI3K family is divided into three different classes (Class I, Class II, and Class III), which are involved in various cellular functions [26, 39]. Class I-PI3K is responsible for the production of phosphatidylinositol-3,4,5-trisphosphate (PI(3,4,5)P3), a second messenger for the translocation and activation of Akt. The PI3K/Akt pathway is known to play important roles in adult neurogenesis. Class III-PI3K (PI3K-III) is responsible for Ptdlns3P production, which recruits downstream effector proteins containing Ptdlns3P-binding domains and is involved in autophagy and endocytic trafficking. Inhibition of class-III PI3K activity by pharmacological treatment or by genetic mutation of PI3K-III complex components has been shown to decrease both the proliferation and differentiation of aNPCs [28, 40], which suggests that the activation of PI3K-III is essential for neurogenesis. In this study, we found that ablation of WDR81 inhibited the proliferation and differentiation of aNPCs by increasing the activity of PI3K-III. Inhibition of PI3K-III activity rescued the proliferation and differentiation deficits of aNPCs in vitro and in vivo caused by loss of WDR81. Thus, the above data suggest that precise regulation of PI3K-III activity and PtdIns3P levels are important for proper neurogenesis in the brain.

Our data revealed that WDR81 inactivation-induced endosomal PtdIns3P elevation acts through the TGFβ signaling pathway to negatively regulate neurogenesis. SARA is a PtdIns3P-binding protein that promotes the recruitment of Smad2 to the ligand-bound TGF-β receptor in the TGFβ signaling pathway [31, 32]. SARA and SARA-related endosomes are known to play important roles in determining cell division of NPCs in the central nervous system [30]. In this research, we found that *Wdr81* deficiency led to persistence of SARA on endosomes, which prevented the degradation of TGFβR and consequently caused hyperactivation of TGFβ signaling that inhibits aNPC proliferation and neuronal differentiation. These findings revealed that WDR81 regulates the SARA-endosome turnover that is required for neurogenesis in the adult brain. Pharmacological inhibition of TGFβ signaling or knockdown of SARA expression in aNPCs markedly rescued the defective neurogenesis in the adult hippocampus of *Wdr81 cKO* mice, suggesting that aberrant SARA-endosome turnover is responsible for the defective neurogenesis in the absence of WDR81. It has been reported previously that TGFβ signaling plays diverse roles in NSC proliferation, neuronal differentiation, and neuronal maturation [35, 41, 42]. SARA acts as an important modulator of these processes by directly interacting with non-activated Smad proteins and TGFβ receptors [31, 32]. Our findings thus provide novel mechanistic insights into the fine-tuning of such signaling by endosomal trafficking.

Mutations of *WDR81* are associated with neurological disorders including CAMRQ2 and microcephaly [16, 17]. Our previous studies revealed that loss of WDR81 and its interaction partner WDR91 disrupted PtdIns3P-dependent endosome conversion in the endosome-lysosome pathway [12, 14]. Here, we further established that the WDR81–SARA–TGFβ axis regulates appropriate adult neurogenesis. Together these findings provide mechanistic explanations for WDR81-associated neurological disorders. In addition, WDR81 can also function independently of PtdIns3P by coordinating with the p62 autophagic receptor to remove ubiquitinated protein aggregates through autophagy [15]. The multifunctional properties of WDR81 suggest that the *WDR81* mutation-associated human diseases could result from defects in diverse cellular processes. Particularly, dysregulation of neurogenesis and neural development are likely responsible for the pathology of *WDR81* mutation-associated CAMRQ2 and microcephaly. Of note, our findings that pharmacological inhibition of PI3K-III and SARA-dependent TGFβ signaling markedly ameliorated the defective neurogenesis caused by *Wdr81* deficiency offer potential therapeutic strategies for treating the mental illness seen in WDR81-associated neurological disorders.

## Electronic supplementary material

Supplemental figures
