# Supplementary material for: WDR81 regulates adult hippocampal neurogenesis through endosomal SARA-TGFβ signaling
Source: Mol Psychiatry. 2018 Dec 7;26(2):694–709. doi: 10.1038/s41380-018-0307-y (PMC7850971; doi:10.1038/s41380-018-0307-y)
Supplement: Supplementary file 1 — Supplemental figures [file 41380_2018_307_MOESM1_ESM.pdf]

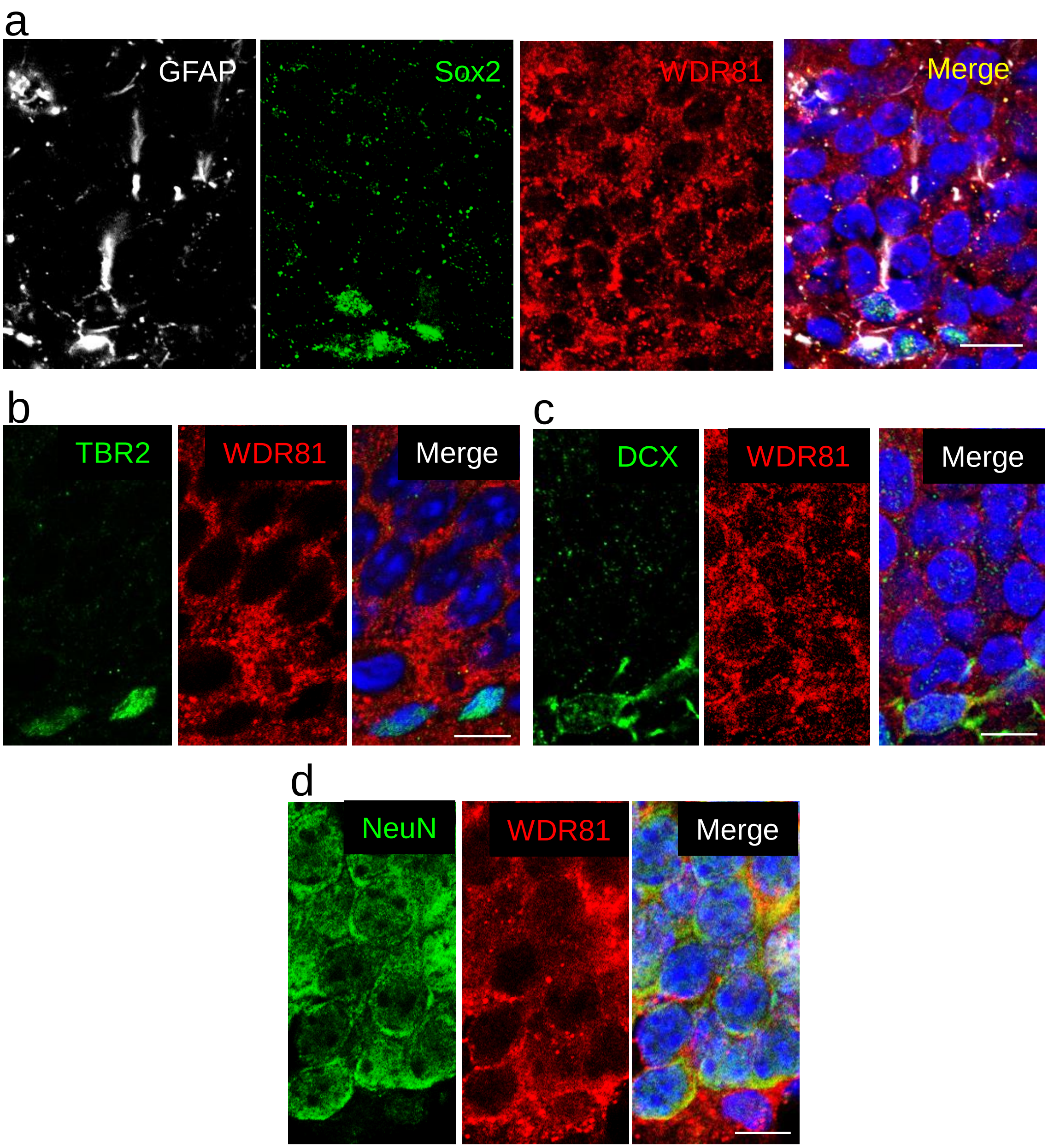

Supplemental Figure 1. WDR81 expression in the adult hippocampus.
(a-d) Immunohistological analysis of WDR81 expression in (a) Sox2^{+}GFAP^{+} type 1 cells and Sox2^{+}GFAP^{-} type 2 cells (Scale bars, 20 µm), as well as in (b) TBR2^{+} progenitor cells, (c) DCX^{+} immature neurons and (d) NeuN^{+} mature neurons in the DG of adult hippocampus (Scale bars, 10 µm).

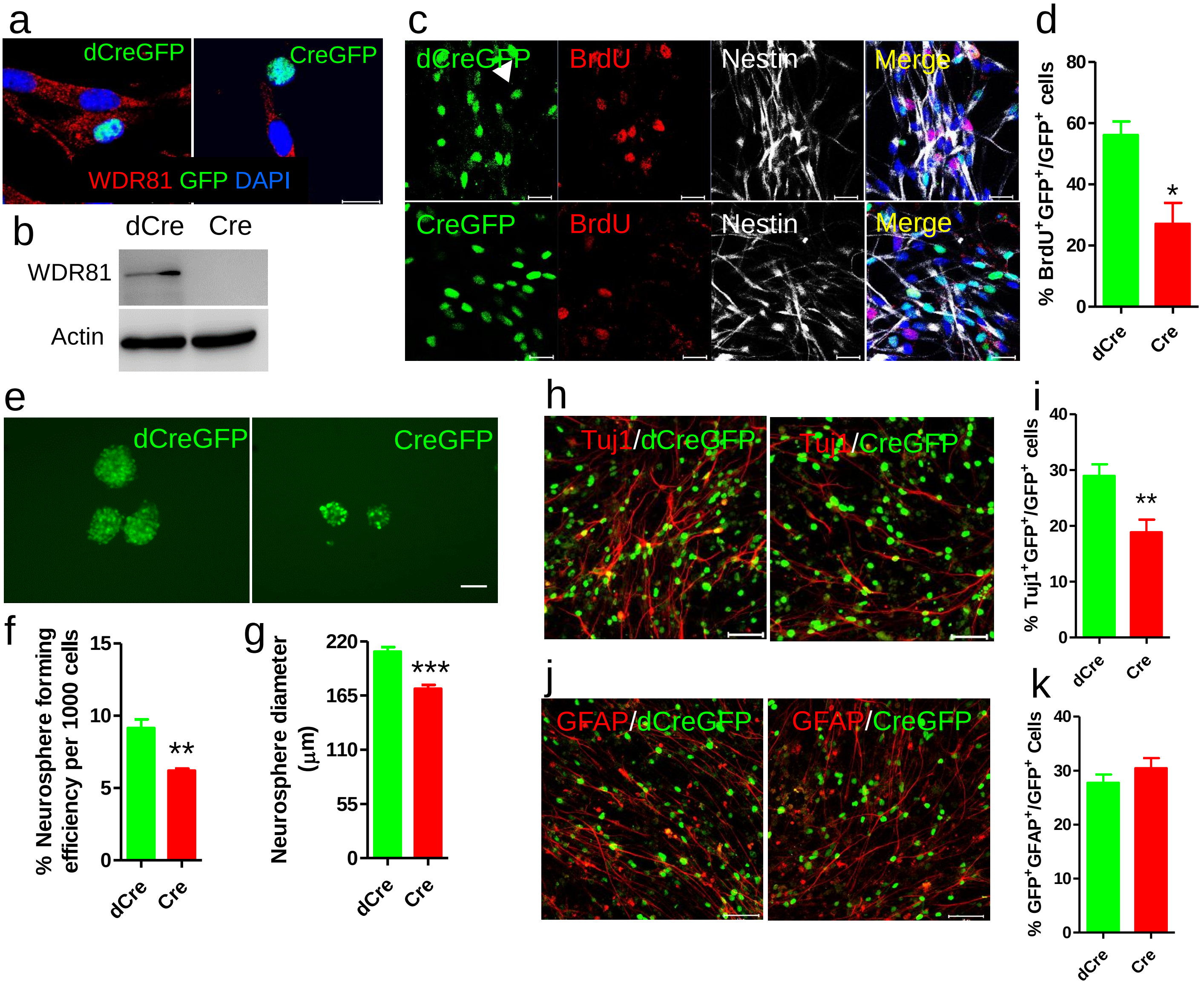

Supplemental Figure 2. WDR81 is required for the proliferation and differentiation of aNPCs *in vitro*.

(a-b) Both immunostaining (a) and western blot (b) analyses of WDR81 expression in *Wdr81*$^{f/f}$ aNPCs infected with lenti-dCreGFP or lenti-CreGFP. Scale bars, 20 μm. (c) Sample images of *Wdr81*$^{f/f}$ aNPCs infected with lenti-dCreGFP or lenti-CreGFP, followed by BrdU pulse labeling and immunocytochemistry analysis. Scale bars, 20 μm. (d) Quantification of BrdU^{+} *Wdr81*$^{f/f}$ aNPCs infected with lenti-dCreGFP or lenti-CreGFP (dCre vs. Cre, t-test, p = 0.023, dCre, n =3 independent experiments, Cre, n = 3 independent experiments). (e) Sample images of *Wdr81*$^{f/f}$ neurospheres infected with lenti-dCreGFP and lenti-CreGFP. Scale bars, 100 μm. (f-g) Quantification of the number and the size of *Wdr81*$^{f/f}$ neurospheres infected with lenti-dCreGFP or lenti-CreGFP (f, number: dCre vs. Cre, t-test, p = 0.008; and g, diameter: WT vs. KO, t-test, p < 0.0001, dCre, n =3 independent experiments, Cre, n = 3 independent experiments). (h) Sample images of differentiated *Wdr81*$^{f/f}$ aNPCs infected with lenti-dCreGFP or lenti-CreGFP, followed by immunostaining with Tuj1, the neuronal marker. Scale bars, 100 μm. (i) Quantification of the percentage of differentiated Tuj1^{+} neurons from *Wdr81*$^{f/f}$ aNPCs infected with lenti-CreGFP or lenti-dCreGFP (dCre vs. Cre, t-test, p = 0.004, dCre, n =3 independent experiments, Cre, n = 3 independent experiments). (j) Sample images of differentiated *Wdr81*$^{f/f}$ aNPCs infected with lenti-dCreGFP or lenti-CreGFP, followed by immunostaining with GFAP, the astrocytic marker. Scale bars, 100 μm. (k) Quantification of differentiated GFAP^{+} astrocytes from *Wdr81*$^{f/f}$ aNPCs infected with lenti-dCreGFP or lenti-CreGFP (dCre vs. Cre, t-test, p = 0.277, dCre, n =3 independent experiments, Cre, n = 3 independent experiments). Data are presented as mean ± SEM; *, p < 0.05; **, p <0.01; ***, p < 0.001.

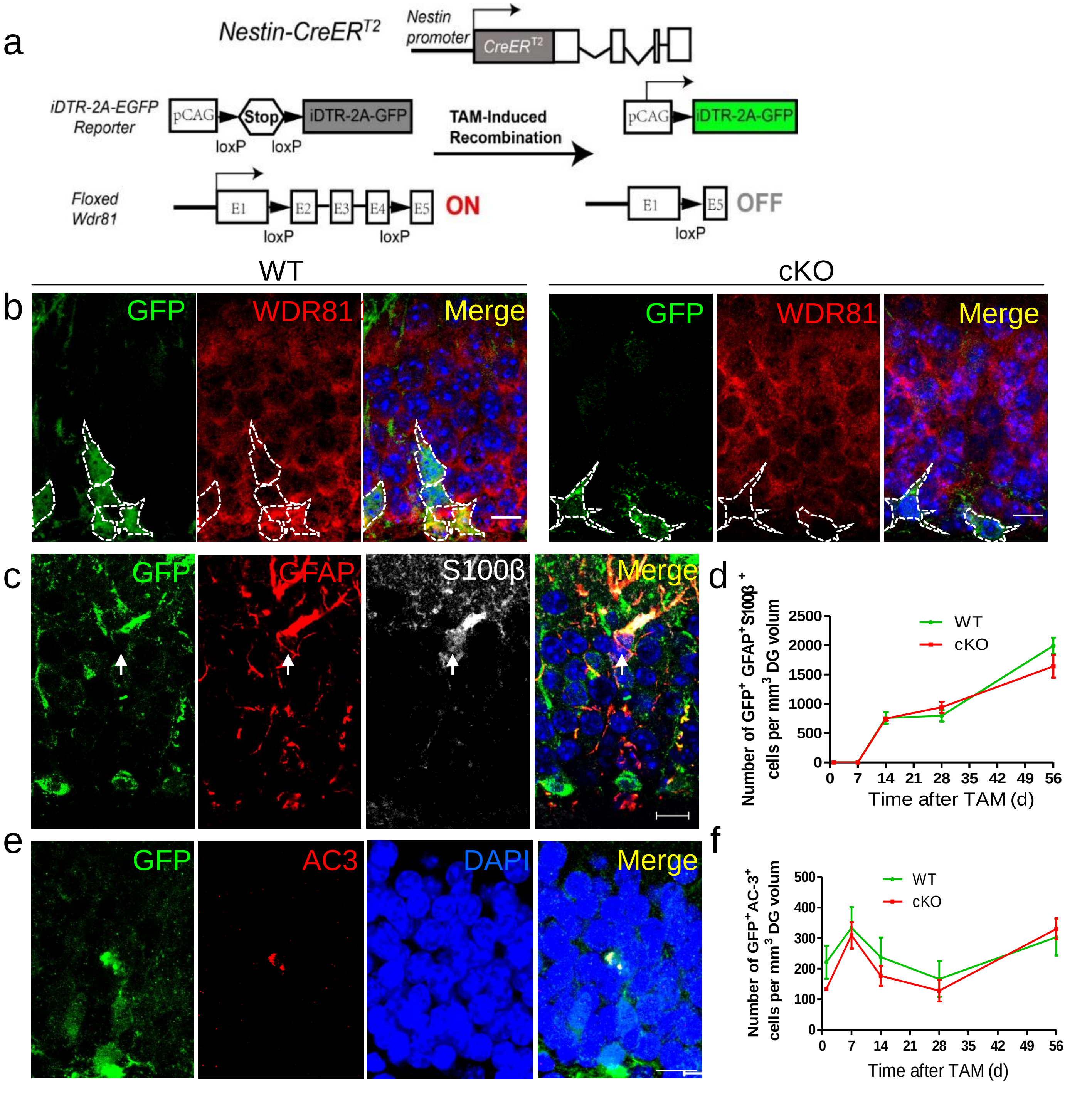

Supplemental Figure 3. Specific ablation of WDR81 from nestin-expressing cells had no effect on astrocyte differentiation and cell apoptosis *in vivo*.

(a) Schematic diagram of *Wdr81* inducible conditional knockout mouse line created by crossing *Wdr81* floxed mice with Nestin-CreERT2 mice and iDTR-2A-GFP mice. (b) Immunohistological analysis of WDR81 expression in the GFP$^+$ cells between WT and *Wdr81 cKO* mice. Scale bars, 10 μm. (c-d) Sample images of GFP$^+$ cells in the DG co-stained with GFAP and S100β (c, Scale bars, 10 μm) and quantification analysis (d) of the number of GFP$^+$S100β$^+$ cells between WT and *Wdr81 cKO* mice at the given time points of post-TAM. (d, 2-way ANOVA, WT vs. cKO, $F_{1,20}$ = 0.563, p = 0.461, WT, n =3 mice, cKO, n = 3 mice). (e-f) Sample images of GFP$^+$ cells in the DG co-stained with activated caspase-3 (AC-3) (e, Scale bars, 10 μm), and quantification analysis (f) of the number of GFP$^+$AC-3$^+$ cells between WT and *Wdr81 cKO* mice at the given time points of post-TAM. (f, 2-way ANOVA, WT vs. cKO, $F_{1,20}$ = 1.41, p = 0.248, WT, n =3 mice, cKO, n = 3 mice). Data are presented as mean ± SEM.

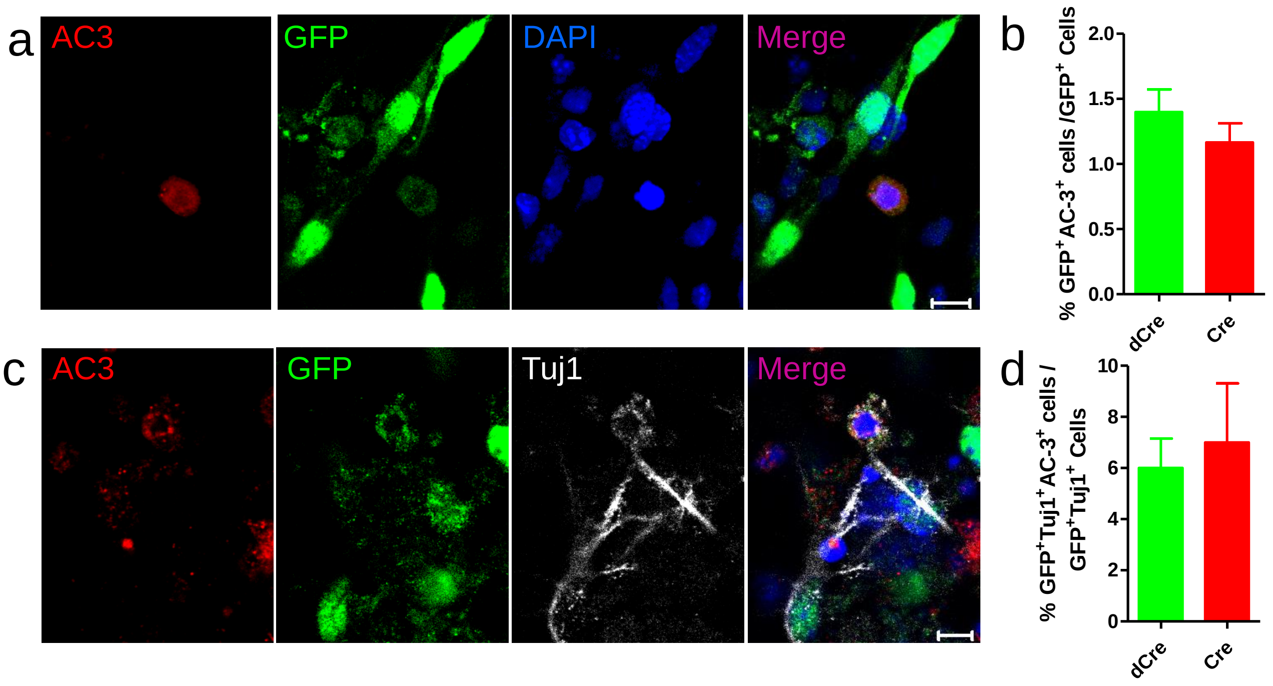

Supplemental Figure 4. WDR81 deficiency did not affect apoptosis of aNPCs *in vitro*

(a) Sample images of *Wdr81*$^{f/f}$ aNPCs infected lenti-CreGFP, followed by immunostaining with activated caspase-3 (AC-3). Scale bars, 10 µm. (b) Quantification analysis of the percentage of AC-3^{+} cells in proliferating *Wdr81*$^{f/f}$ aNPCs infected with lenti-dCreGFP or lenti-CreGFP (dCre vs. Cre, t-test, $p = 0.36$, dCre, $n = 3$ independent experiments, Cre, $n = 3$ independent experiments). (c) Sample images of differentiated *Wdr81*$^{f/f}$ aNPCs infected with lenti-dCreGFP or lenti-CreGFP, followed by immunostaining with Tuj1 and activated caspase-3 (AC-3). Scale bars, 10 µm. (d) Quantification analysis of the percentage of AC-3^{+} cells in differentiated Tuj1^{+} cells from *Wdr81*$^{f/f}$ aNPCs infected with lenti-dCreGFP or lenti-CreGFP (dCre vs. Cre, t-test, $p = 0.718$, dCre, $n = 3$ independent experiments, Cre, $n = 3$ independent experiments). Data are presented as mean ± SEM.

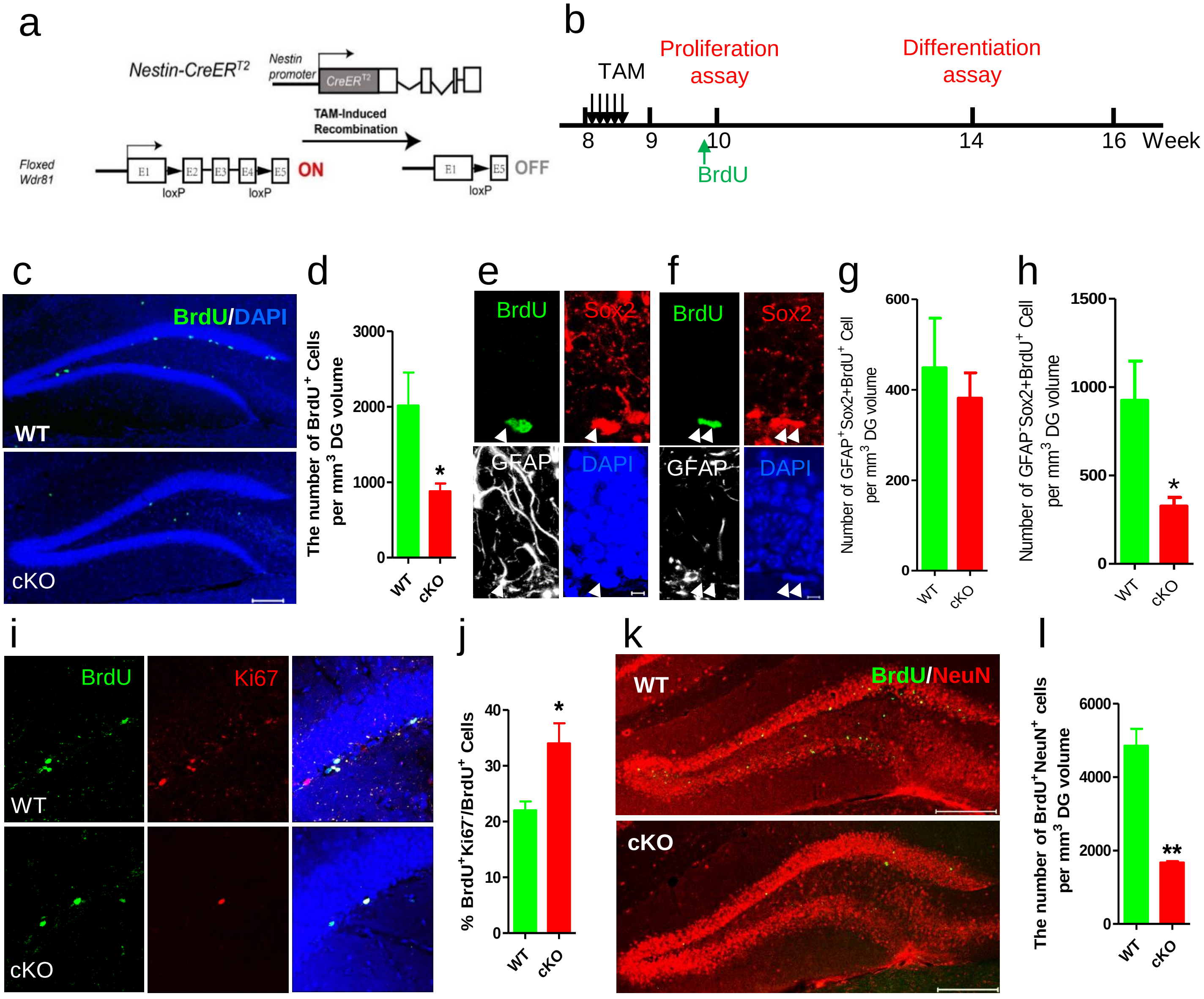

Supplemental Figure 5. WDR81 deficiency led to promoted cell cycle exit of aNPCs and reduced neurogenesis *in vivo*.

(a) Schematic diagram of *Wdr81* inducible conditional knockout mouse line created by crossing *Wdr81* floxed mice only with Nestin-CreERT2 mice. (b) Experimental scheme for assessing the proliferation and differentiation of aNPC in the DG of adult hippocampus. (c) Sample images of $BrdU^+$ cells in the DG of WT and *Wdr81 cKO* mice at 2 hours post BrdU injection. Scale bars, 100 µm. (d) Quantification of $BrdU^+$ cells in the DG of *Wdr81 cKO* mice and WT mice (WT vs. cKO, t-test, $p = 0.029$, WT, n = 6 mice, cKO, n = 6 mice) at 2 hours post BrdU labeling. (e-f) Sample images of $BrdU^+Sox2^+GFAP^+$ (e) and $BrdU^+Sox2^+GFAP^-$ (f) cells in the DG. Scale bars, 10 µm. (g-h) Quantification analysis of the number of $BrdU^+GFAP^+Sox2^+$ cells (g, WT vs. cKO, t-test, $p = 0.665$, WT, n = 5 mice, cKO, n = 5 mice) and $BrdU^+GFAP^-Sox2^+$ cells (h, WT vs. cKO, t-test, $p = 0.03$, WT, n = 5 mice, cKO, n = 5 mice) between WT and *Wdr81 cKO* mice at 2-hours post BrdU injection. (i) Sample images of $BrdU^+Ki67^+$ cells 24 hours post BrdU injection. Scale bars, 50 µm. (j) Ratio of $BrdU^+Ki67^-$ cells among total $BrdU^+$ cells in the DG of *Wdr81 cKO* mice and WT mice at 24 hours post BrdU labeling (WT vs. cKO, t-test, $p = 0.019$, WT, n = 4 mice, cKO, n = 3 mice). (k) Sample images of $BrdU^+NeuN^+$ cells in the DG of WT and *Wdr81 cKO* mice at 4 weeks post BrdU injection. Scale bars, 200 µm. (l) Quantification of $BrdU^+$ cells in the DG of *Wdr81 cKO* mice and WT mice (WT vs. cKO, t-test, $p = 0.0024$, WT, n = 3 mice, cKO, n = 3 mice). Data are presented as mean ± SEM; *, $p < 0.05$; **, $p < 0.01$.

a

b

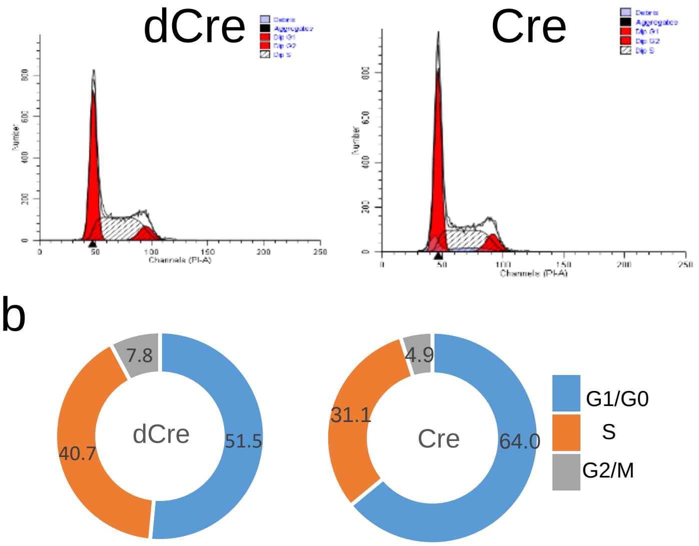

Supplemental Figure 6. WDR81 deficiency led to impaired cell cycle of aNPCs *in vitro*. (a) DNA content and cell cycle analysis of cultured *Wdr81*$^{f/f}$ aNPCs infected either lenti-dCreGFP or lenti-CreGFP. (b) Quantification analysis of the percentage of *Wdr81*$^{f/f}$ aNPCs, infected lenti-dCreGFP or lenti-CreGFP, in the G1/G0 phase (dCre vs. Cre, t-test, $p = 0.009$) and the S phase (dCre vs. Cre, t-test, $p = 0.006$). dCre, $n = 3$ independent experiments, Cre, $n = 3$ independent experiments. Data are presented as mean ± SEM.

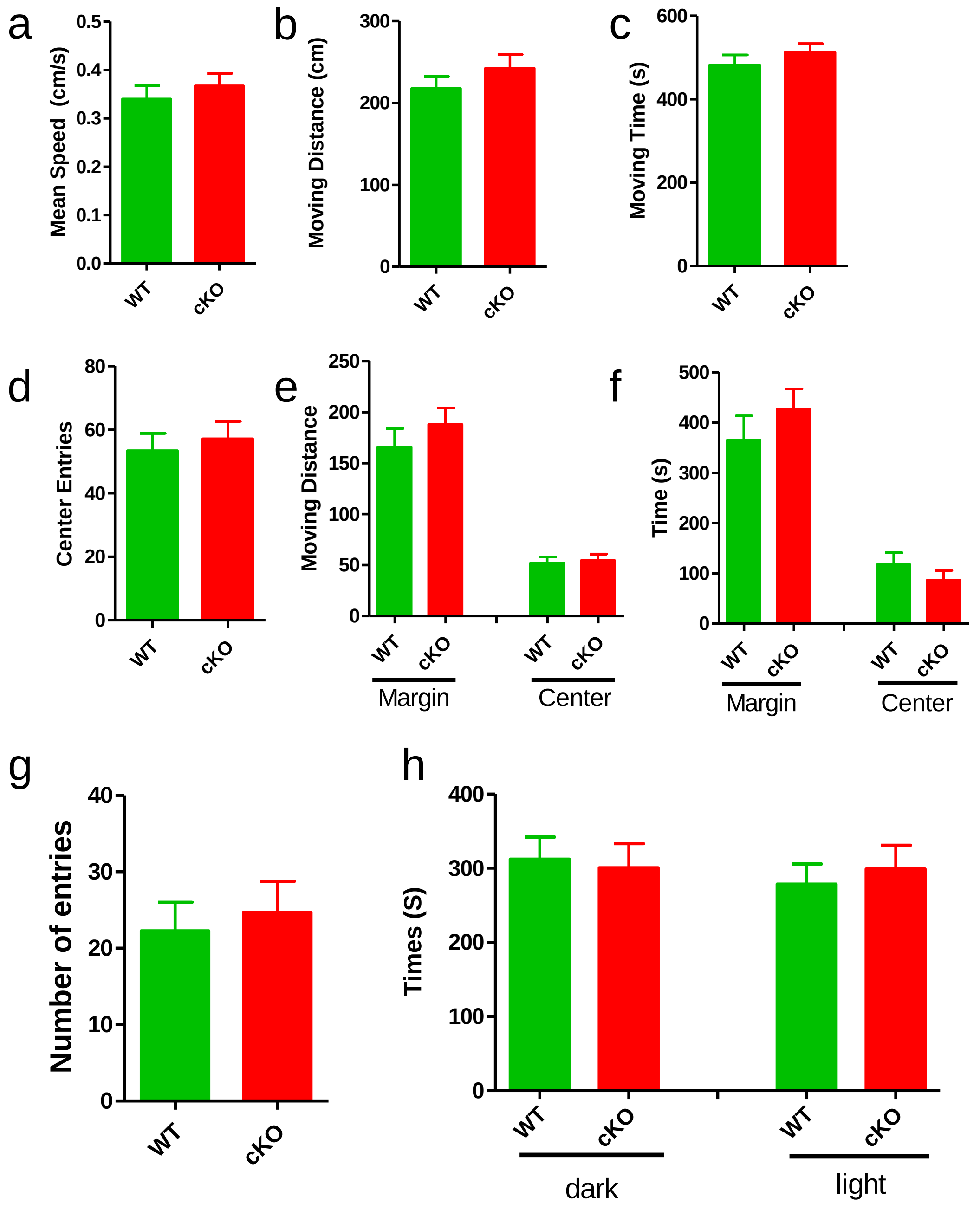

Supplemental Figure 7. *Wdr81 cKO* mice are not deficient in open-field and dark/light exploration tests.

(a-c), There was no difference in the mean moving speed (a, WT vs. cKO, t-test, $p = 0.483$), the total distance traveled (b, WT vs. cKO, t-test, $p = 0.288$), or the total time spent moving (c, WT vs. cKO, t-test, $p = 0.337$) in the floor plane between WT and *Wdr81 cKO* mice in the open-filed task. (d-f), The *Wdr81 cKO* mice did not exhibit reduced exploration in the center of the arena, shown as the number of entries to the center (d, WT vs. cKO, t-test, $p = 0.64$), the distance traveled (e, margin: WT vs. cKO, t-test, $p = 0.38$; center: WT vs. cKO, t-test, $p = 0.776$), or the time spent in the center of the arena (f, margin: WT vs. cKO, t-test, $p = 0.337$; center: WT vs. cKO, t-test, $p = 0.336$) in the open-filed task. (g-h) There was no difference in the total entries (g, WT vs. cKO, t-test, $p = 0.666$) and the time spent in the dark/light compartment (h, dark: WT vs. cKO, t-test, $p = 0.795$; light: WT vs. cKO, t-test, $p = 0.64$) between WT and *Wdr81 cKO* mice during the dark/light exploration test. WT, n =7 mice, cKO, n = 7 mice. Data are presented as mean ± SEM.

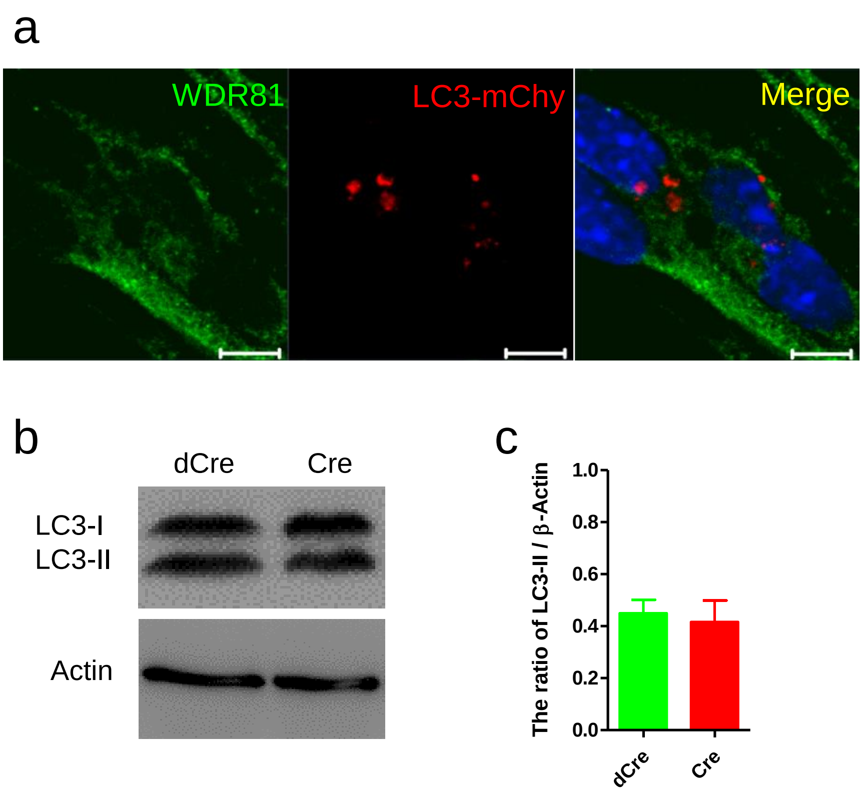

Supplemental Figure 8. WDR81 deficiency did not affect general autophagy in aNPCs.
(a) WDR81 did not coolcolized with LC3-mCherry positive autophagosome in aNPCs. Scale bars, 5 µm. (b and c) Western blot analysis of LC3-I and II expression in *Wdr81*$^{f/f}$ aNPCs infected with lenti-dCreGFP or lenti-CreGFP (b) and quantification of the ratio of LC3-II/Actin (c, dCre vs. Cre, t-test, $p = 0.74$; dCre, $n = 3$ independent experiments, Cre, $n = 3$ independent experiments). Data are presented as mean ± SEM.

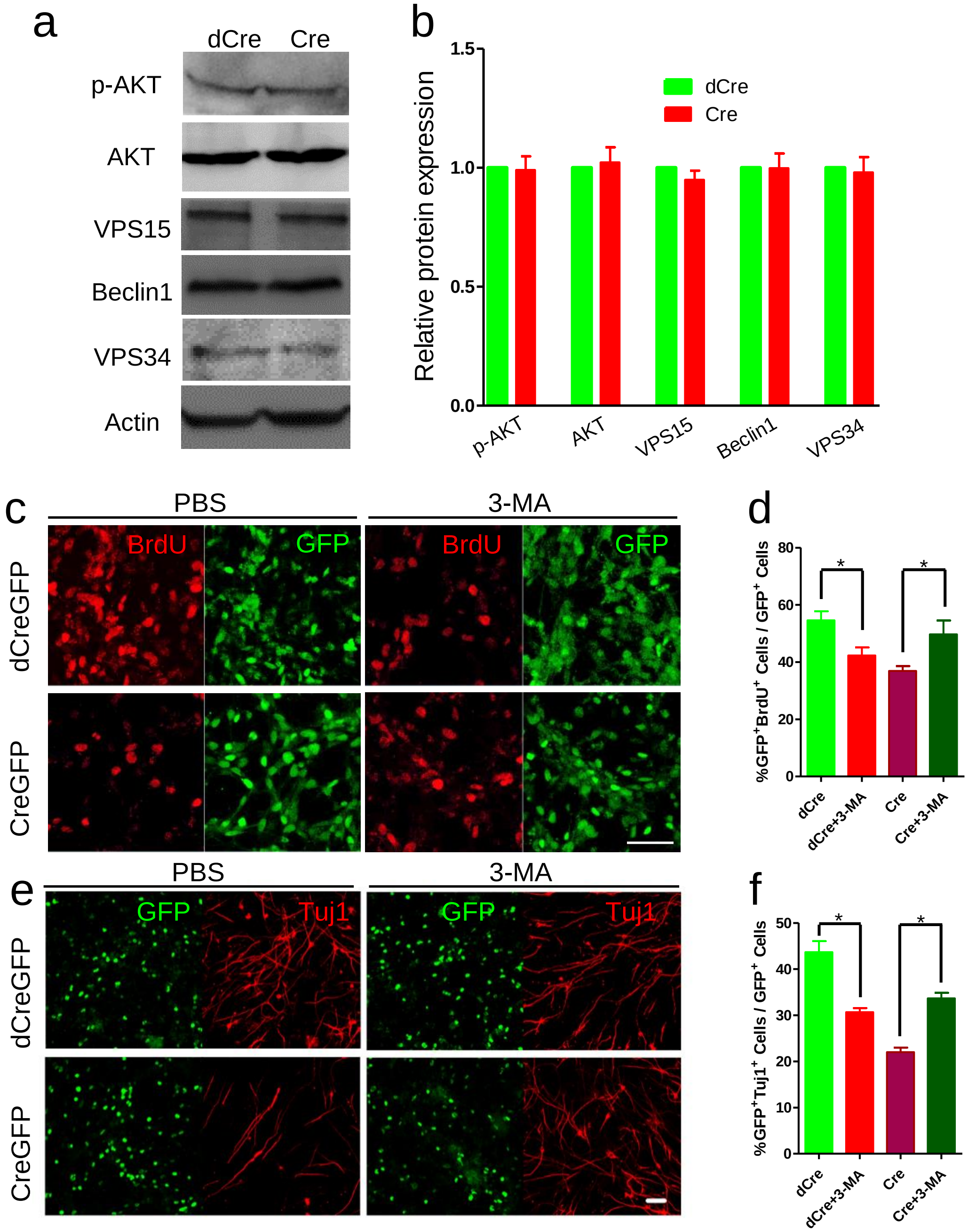

Supplemental Figure 9. Inhibition of PI3K-III activity rescued WDR81-deficient aNPCs proliferation and differentiation *in vitro*.

(a-b) Western blot analysis (a) and quantification (b) of p-AKT, AKT, VPS15, Beclin1 and VPS34 expression in *Wdr81*$^{f/f}$ aNPCs infected with lenti-dCreGFP or lenti-CreGFP. dCre, n = 3 independent experiments, Cre, n = 3 independent experiments. (c) Sample images of *Wdr81*$^{f/f}$ aNPCs infected with lenti-dCreGFP or lenti-CreGFP, followed by 3-MA treatment and BrdU pulse labeling. Scale bars, 50 μm. (d) Quantification of BrdU^{+} cells in *Wdr81*$^{f/f}$ aNPCs infected with lenti-CreGFP or lenti-dCreGFP and treated with 3-MA (dCre vs. dCre+3-MA, t-test, $p = 0.013$, and Cre vs. Cre+3-MA, t-test, $p = 0.024$). dCre, n =3 independent experiments, dCre+3-MA, n =3 independent experiments, Cre, n = 3 independent experiments, Cre+3-MA, n =3 independent experiments. (e) Sample images of *Wdr81*$^{f/f}$ aNPCs infected with lenti-dCreGFP or lenti-CreGFP, followed by 3-MA treatment and Tuj1^{+} staining. Scale bars, 50 μm. (f) Quantification of differentiated Tuj1^{+} cells from *Wdr81*$^{f/f}$ aNPCs infected with lenti-dCreGFP or lenti-CreGFP and followed by treatment with 3-MA (dCre vs. dCre+3-MA, t-test, $p = 0.007$, and Cre vs. Cre+3-MA, t-test, $p = 0.002$). dCre, n =3 independent experiments, dCre+3-MA, n =3 independent experiments, Cre, n = 3 independent experiments, Cre+3-MA, n =3 independent experiments. Data are presented as mean ± SEM; *, $p < 0.05$.

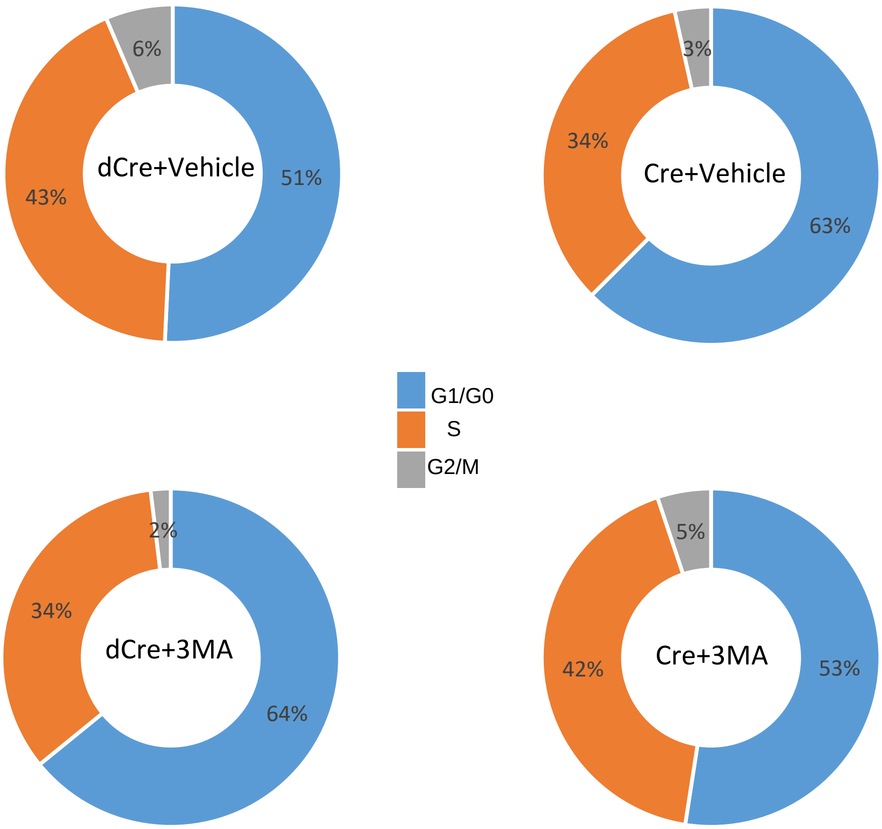

Supplemental Figure 10. Inhibition of PI3K-III activity rescued cell cycle defects of WDR81-deficient aNPCs *in vitro*.

Quantification analysis of the percentage of *Wdr81*$^{f/f}$ aNPCs, infected lenti-dCreGFP or lenti-CreGFP and treated with 3-MA, in the G1/G0 phase (Cre+vehicle vs. Cre+3MA, t-test, p = 0.02) and the S phase (Cre+vehicle vs. Cre+3MA, t-test, p = 0.009). dCre+vehicle, n = 3 independent experiments, Cre+vehicle, n = 3 independent experiments, dCre+3MA, n = 3 independent experiments, Cre+3MA, n = 3 independent experiments. Data are presented as mean ± SEM.

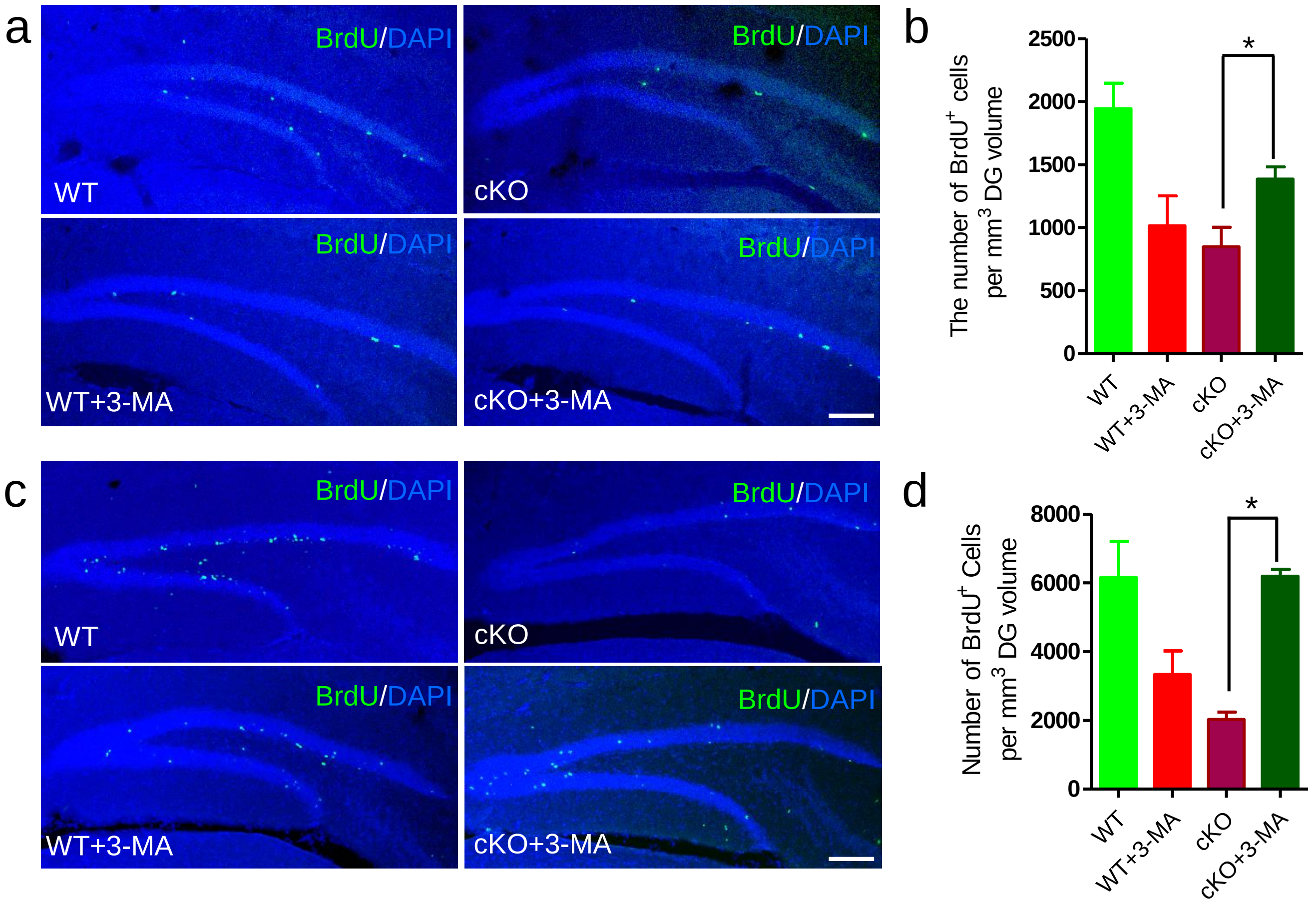

Supplemental Figure 11. Inhibition of PI3K-III activity rescued adult hippocampal neurogenesis defects in *WDR81 cKO* mice.

(a) Sample images of BrdU$^+$ cells in the DG of WT and *Wdr81 cKO* mice with or without 3-MA treatment post 2-hours BrdU injection. Scale bars, 100 µm. (b) Quantification analysis of the number of BrdU$^+$ cells in the DG of WT and *Wdr81 cKO* mice with or without 3-MA treatment post 2-hours BrdU injection (WT vs. WT+3-MA, t-test, $p = 0.019$; cKO vs. cKO+3-MA, t-test, $p = 0.019$). (c) Sample images of BrdU$^+$ cells in the DG of WT and *Wdr81 cKO* mice with or without 3-MA treatment post 4-weeks BrdU injection. Scale bars, 100 µm. (d) Quantification analysis of the number of BrdU$^+$ cells in the DG of WT and *Wdr81 cKO* mice with or without 3-MA treatment post 4-weeks BrdU injection (WT vs. WT+3-MA, t-test, $p = 0.033$; cKO vs. cKO+3-MA, t-test, $p = 0.049$). WT, n = 5 mice, WT+3-MA, n = 4 mice, cKO, n = 5 mice, cKO+3-MA, n =5 mice. Data are presented as mean ± SEM; *, $p < 0.05$.

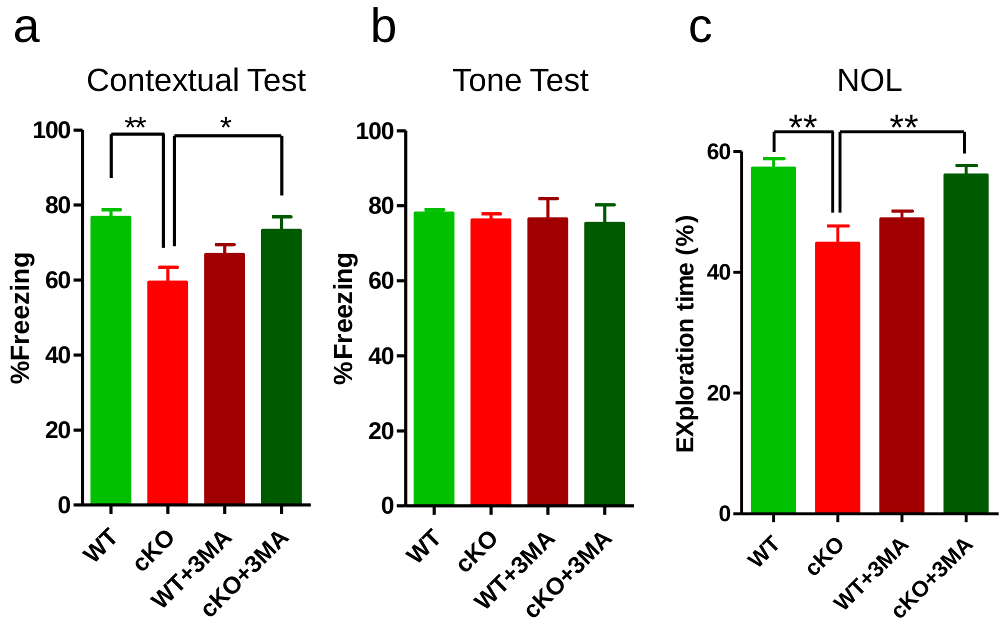

Supplemental Figure 12. Inhibition of PI3K-III activity rescued the deficits of hippocampal dependent learning in *WDR81 cKO* mice.

(a-b) Analysis of the percentage of freezing behavior in WT and *Wdr81 cKO* mice treated with 3-MA during fear conditioning tests (c, the contextual test, cKO vs. cKO+3-MA, t-test, $p = 0.024$; d, the tone test, cKO vs. cKO+3-MA, t-test, $p = 0.87$). (c) Analysis of the percentage of exploration time on novel objective location in WT and *Wdr81 cKO* mice treated with 3-MA (cKO vs. cKO+3-MA, t-test, $p = 0.004$). WT, n = 7 mice, cKO, n = 7 mice, WT+3-MA, n = 7 mice, cKO+3-MA, n = 7 mice. Data are presented as mean ± SEM; *, $p < 0.05$; **, $p <0.01$; ***, $p < 0.001$.

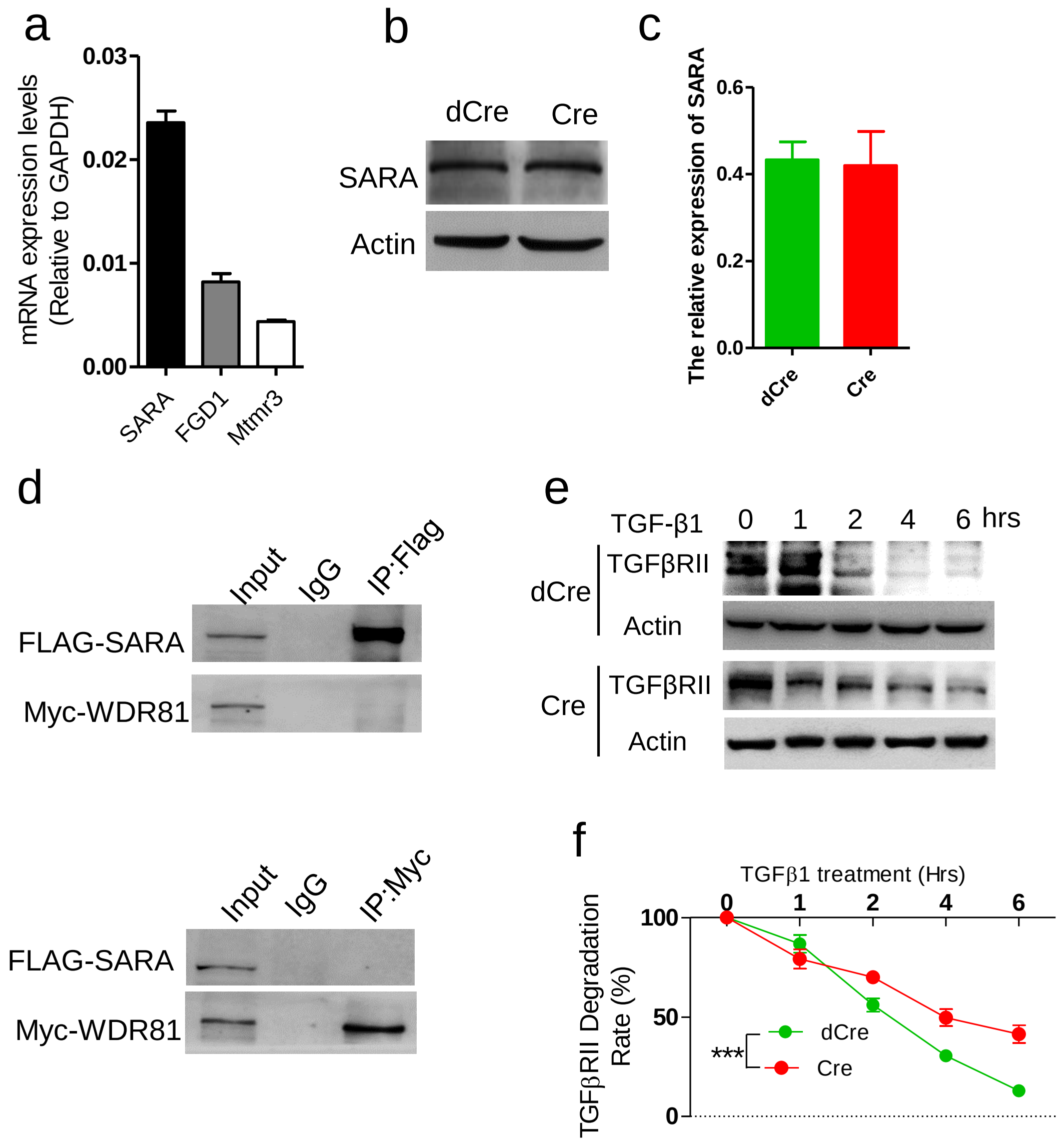

Supplemental Figure 13. WDR81 regulated SARA-mediated TGFβ signaling.
(a) Real-time RT-PCR analysis of the expression levels of SARA, FGD1 and Mtmr3 in aNPCs. (b-c) Western blot analysis (b) and quantification (c) of SARA expression in *Wdr81$^{f/f}$* aNPCs infected with lenti-dCreGFP or lenti-CreGFP (dCre, n = 3 independent experiments, Cre, n = 3 independent experiments, t-test, dCre vs. Cre, p = 0.887). (d) Co-IP analysis of FLAG-SARA and Myc-WDR81. (e) Western blot analysis of TGFβRII expression levels in *Wdr81$^{f/f}$* aNPCs infected with lenti-dCreGFP or lenti-CreGFP after treating with TGFβ1 at the indicated time points. (f) Quantification analysis of TGFβRII expression levels with imageJ software (dCre, n = 3 independent experiments, Cre, n = 3 independent experiments; 2-way ANOVA, significant effect of genotype, $F_{1,20}$ = 3.6, $p < 0.0001$). Data are presented as mean ± SEM; ***, $p < 0.001$.

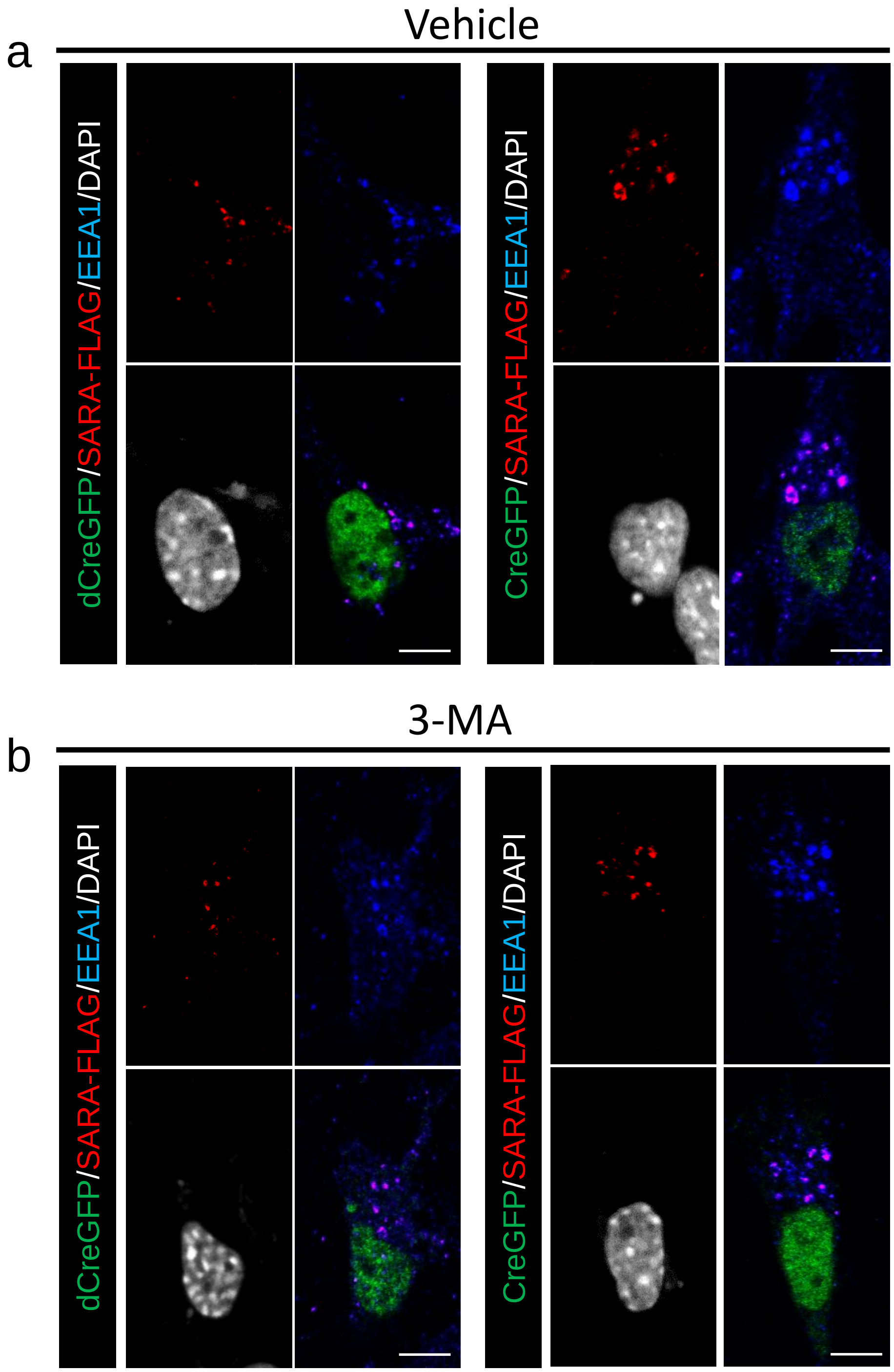

Supplemental Figure 14. Inhibition of PI3K-III activity rescued the aberrant SARA endosome in WDR81 deficient aNPCs.

(a-b) Co-staining of SARA with EEA1 in Wdr81f/f aNPCs infected with either lenti-dCreGFP or lenti-CreGFP and treated with or without 3-MA. Scale bars, 5 µm.

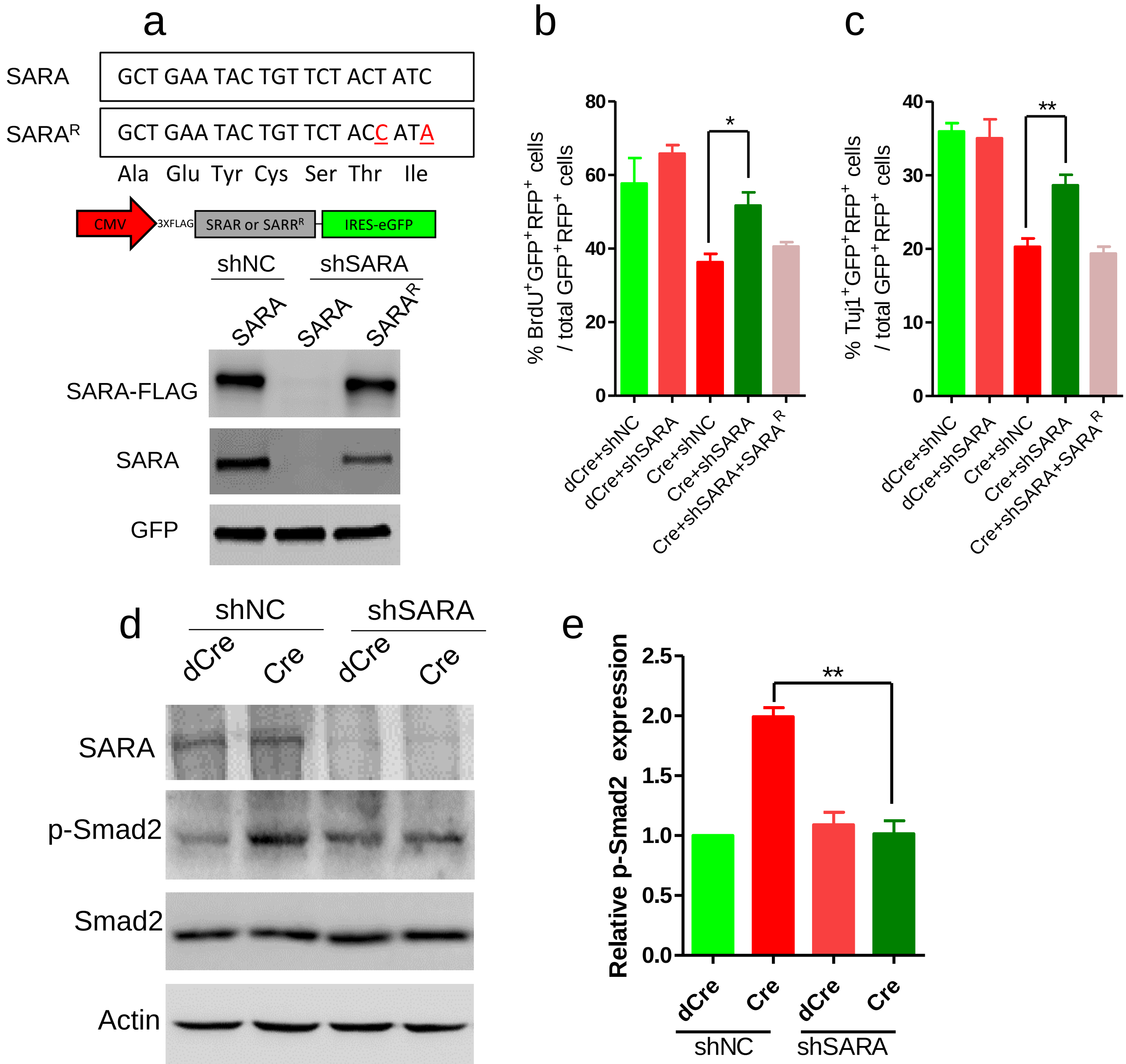

Supplemental Figure 15. Inhibition of SARA-mediated TGFβ signaling rescued the defects of WDR81-deficient aNPCs *in vitro*.

(a) Alignment of shSARA targeting sequence in SARA and silent mutations made in SARA^R (underlined), and western blot analysis of the efficacy and specificity of shRNA against mouse *SARA*. (b) Quantification analysis of the percentage of BrdU$^+$ *Wdr81$^{f/f}$* aNPCs co-infected with lenti-dCreGFP or lenti-CreGFP with shRNA lenti-NC-shRNA or lenti-SARA-shRNA. (Cre+shNC vs. Cre+shSARA, T-tetst, $p = 0.022$. dCre+shNC: n =3 independent experiments, dCre+shSARA, n =3 independent experiments, Cre+shNC, n = 3 independent experiments, Cre+shSARA, n =3 independent experiments, Cre+shSARA+SARA^R, n =3 independent experiments). (c) Quantification analysis of the percentage of differentiated Tuj1$^+$ cells from Wdr81$^{f/f}$ aNPCs co-infected with lenti-dCreGFP or lenti-CreGFP with shRNA lenti-NC-shRNA or lenti-SARA-shRNA. (Cre+shNC vs. Cre+shSARA, t-test, $p = 0.001$; dCre+shNC: n =3 independent experiments, dCre+shSARA, n =3 independent experiments, Cre+shNC, n = 3 independent experiments, Cre+shSARA, n =3 independent experiments, Cre+shSARA+SARA^R, n =3 independent experiments). (d-e) Western blot analysis and quantification of p-Smad2 expression in *Wdr81$^{f/f}$* aNPCs co-infected the lenti-dCreGFP or lenti-CreGFP with lenti-NC-shRNA or lenti-SARA-shRNA (e, Cre+shNC v.s. Cre+shSARA, t-test, $p = 0.0018$; dCre+shNC, n =3 independent experiments, dCre+shSARA, n = 3 independent experiments, Cre+shNC, n = 3 independent experiments, Cre+shSARA, n = 3 independent experiments. Data are presented as mean ± SEM; *, $p < 0.05$; **, $p < 0.01$.

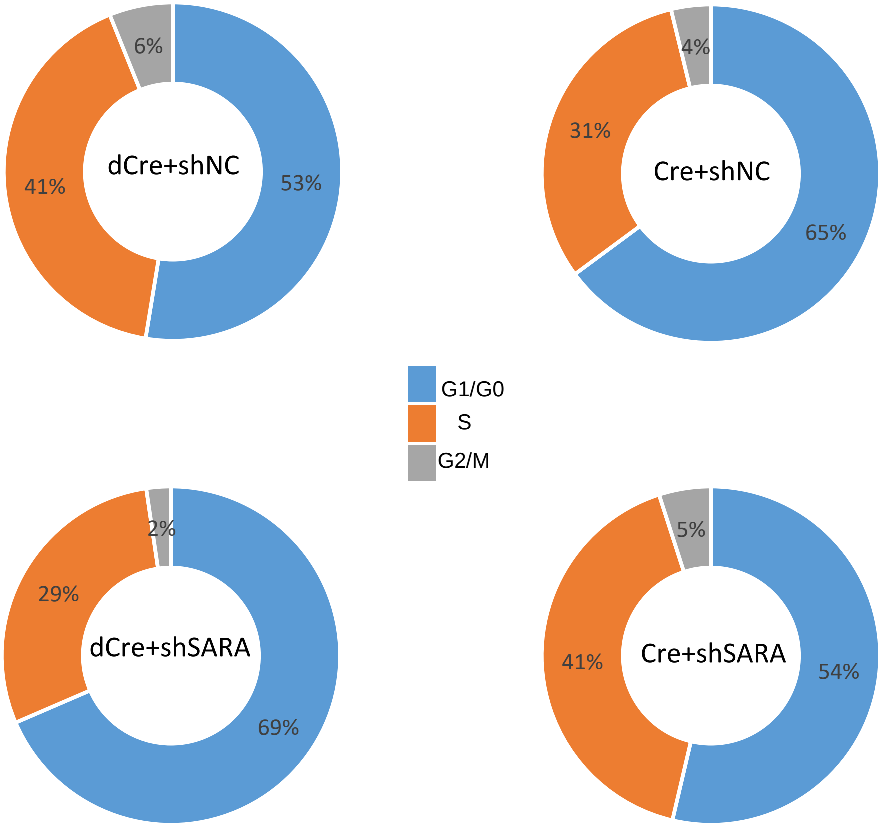

Supplemental Figure 16. Inhibition of SARA-mediated TGFβ signaling rescued cell cycle defects of WDR81-deficient aNPCs *in vitro*.

(a) Quantification analysis of the percentage of *Wdr81*$^{f/f}$ aNPCs, infected lenti-dCreGFP or lenti-CreGFP and treated with LY, in the G1/G0 phase (Cre+shNC vs. Cre+shSARA, t-test, $p = 0.0001$) and the S phase (Cre+shNC vs. Cre+shSARA, t-test, $p = 0.0002$). dCre+shNC, n = 3 independent experiments, dCre+shSARA, n = 3 independent experiments, Cre+shNC, n = 3 independent experiments, Cre+shSARA, n = 3 independent experiments. Data are presented as mean ± SEM.

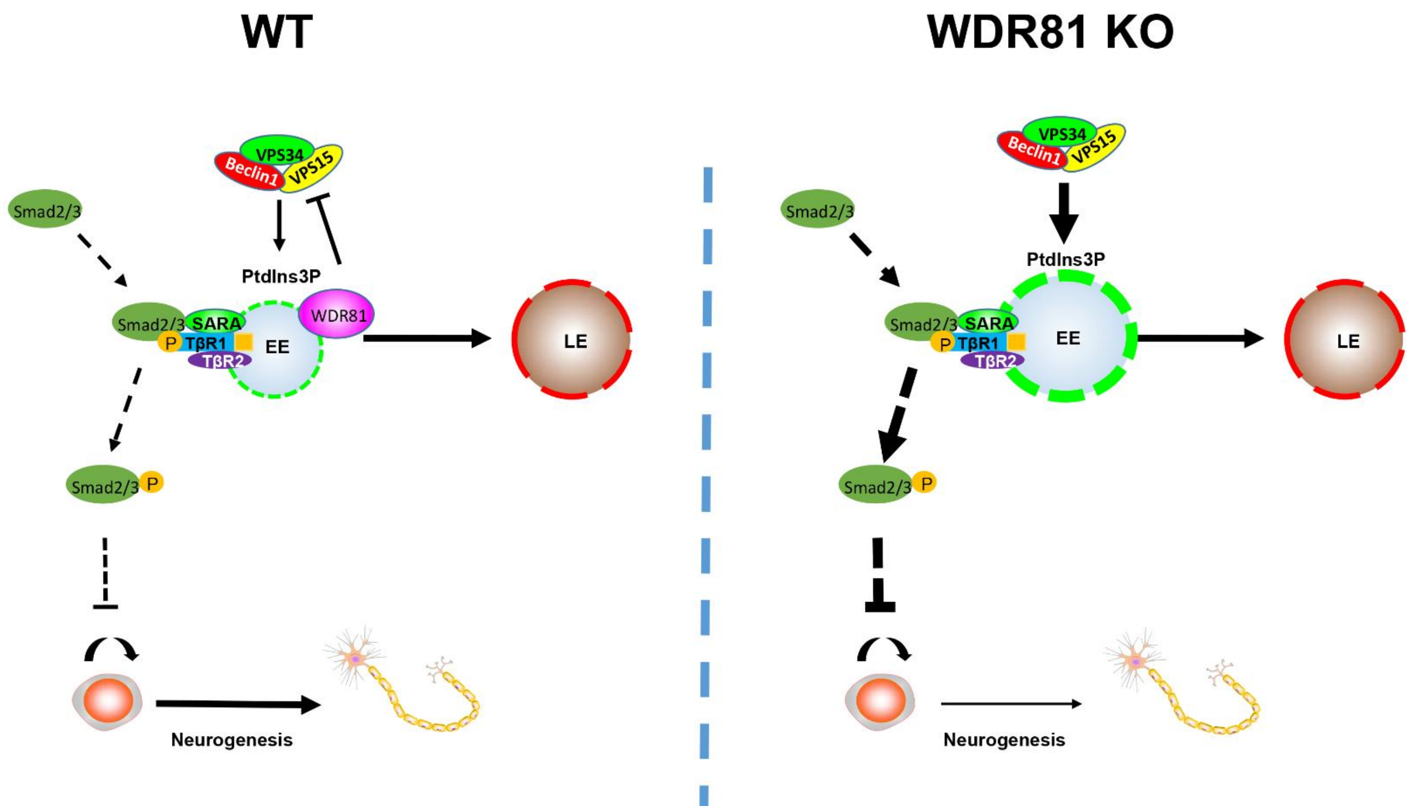

Supplemental Figure 17. Working model for underlying mechanism of WDR81 regulating adult neurogenesis through endosomal SARA-TGFβ signaling.
